# Nuclear‐Localized BCKDK Facilitates Homologous Recombination Repair to Support Breast Cancer Progression and Therapy Resistance

**DOI:** 10.1002/advs.202416590

**Published:** 2025-04-29

**Authors:** Haiying Liu, Jiaqian Feng, Tingting Pan, Pinggen Zhang, Ling Ye, Zetan Jiang, Zilong Zhou, Qiankun Mao, Jian Li, Xinyi Yang, Ping Gao, De Huang, Huafeng Zhang

**Affiliations:** ^1^ Department of General Surgery The First Affiliated Hospital of USTC Division of Life Science and Medicine University of Science and Technology of China Hefei 230027 China; ^2^ Key Laboratory of Immune Response and Immunotherapy School of Basic Medical Sciences Division of Life Science and Medicine University of Science and Technology of China Hefei 230027 China; ^3^ Medical Research Institute Guangdong Provincial People‘s Hospital Guangdong Academy of Medical Sciences Southern Medical University Guangzhou 510080 China; ^4^ Center for Advanced Interdisciplinary Science and Biomedicine of IHM Division of Life Sciences and Medicine University of Science and Technology of China Hefei 230001 China; ^5^ Institute of Health and Medicine Hefei Comprehensive National Science Center Hefei 230601 China; ^6^ Anhui Key Laboratory of Molecular Oncology Hefei 230026 China

**Keywords:** BCKDK, breast cancer, combinational therapy, DNA repair inhibitor, homologous recombination repair

## Abstract

Homologous recombination repair (HRR) is crucial for maintaining genomic stability by repairing DNA damage. Despite its importance, HRR's role in cancer progression is not fully elucidated. Here, this work shows that nuclear‐localized branched‐chain α‐ketoacid dehydrogenase kinase (BCKDK) acts as a modulator of HRR, promoting cell resistance against DNA damage‐inducing therapy in breast cancer. Mechanistically, this work demonstrates that BCKDK is localized in the nucleus and phosphorylates RNF8 at Ser157, preventing the ubiquitin‐mediated degradation of RAD51, thereby facilitating HRR‐mediated DNA repair under replication stress. Notably, aberrant expression of the BCKDK/p‐RNF8/RAD51 axis correlates with breast cancer progression and poor patient survival. Furthermore, this work identifies a small molecule inhibitor of BCKDK, GSK180736A, that disrupts its HRR function and exhibits strong tumor suppression when combined with DNA damage‐inducing drugs. Collectively, this study reveals a new role of BCKDK in regulating HRR, independent of its metabolic function, presenting it as a potential therapeutic target and predictive biomarker in breast cancer.

## Graphical abstract

Under DNA damage, tumor cells rely on efficient DNA repair for survival and therapy resistance. This study has demonstrated that BCKDK localizes to breast cancer cell nuclei, where it binds to and phosphorylates RNF8, thereby blocking ubiquitin‐mediated degradation of RAD51 and enhancing HRR. A selective BCKDK inhibitor synergizes with clinical agents, suggesting a promising therapeutic strategy for breast cancer.

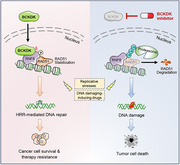

## 1 Introduction

To preserve genome integrity, cells have developed homologous recombination repair (HRR), which is essential for the repair of DNA double‐strand breaks and for the protection of stalled replication forks.^[^ ^1^, ^2^, ^3^ ^]^ The increasing accumulation of DNA damage observed during the pre‐ or early stages of cancer, often due to replication stress, suggests that HRR acts as a replication escort to prevent cancer initiation.^[^ ^4^, ^5^ ^]^ In certain types of cancers,^[^ ^6^, ^7^, ^8^ ^]^ mutations in HRR‐related genes, such as *BRCA* mutations leading to homologous recombination deficiency, which provide promising therapeutic strategies for cancer treatment. Among them, poly‐ADP ribose polymerase (PARP) inhibitors have demonstrated significant efficacy in the treatment of cancers harboring HRR gene mutations. These cancer cells exhibit an increased reliance on PARP for the repair of DNA damage due to their inherent defects. Clinical administration of PARP inhibitors has yielded substantial therapeutic benefits in several cancer types, including breast cancer, ovarian cancer, prostate cancer, and pancreatic cancer.^[^ ^9^, ^10^, ^11^, ^12^ ^]^ In addition to the success of these DNA repair inhibitors in treating triple‐negative breast cancer (TNBC) patients with *BRCA* mutations, resistance to PARP inhibitors in both *BRCA*‐mutated and wild‐type tumors has emerged as a significant limitation and therapeutic challenge in clinical cancer treatments.^[^ ^13^, ^14^, ^15^, ^16^ ^]^

Despite the role of HRR in suppressing genomic instability‐mediated tumorigenesis, enhanced HRR activity can facilitate tumor cell survival by repairing DNA damage incurred as a result of high levels of replication stress, thereby supporting continuous proliferation.^[^ ^17^, ^18^, ^19^ ^]^ This is particularly evident in cancers with mutations in other DNA repair pathways, where HRR becomes a critical survival mechanism.^[^ ^20^ ^]^ For example, elevated levels of recombinase RAD51, an important regulator of HRR that forms a nucleoprotein filament on resected single‐stranded DNA at the damage site,^[^ ^21^, ^22^, ^23^, ^24^, ^25^ ^]^ promote cancer cell proliferation, and may confer resistance to DNA‐targeting treatments. This can lead to the survival of cancer cells and poor prognosis.^[^ ^22^, ^26^, ^27^, ^28^ ^]^ Thus, tumor cell‐intrinsic compensatory DNA repair pathways could confer resistance to the PARP inhibition approaches.^[^ ^29^, ^30^, ^31^ ^]^ Given the dual role of HRR in regulating cancer progression, it is imperative to enhance the mechanistic understanding of HRR to fully validate HRR sub‐pathways as relevant targets in cancer therapy.

Branched‐chain α‐ketoacid dehydrogenase kinase (BCKDK) is a pivotal enzyme known for its role in regulating the rate‐limiting and irreversible step of branched‐chain amino acid (BCAA) catabolism. As a kinase, BCKDK phosphorylates the branched‐chain α‐ketoacid dehydrogenase complex (BCKDH complex), thereby reducing its activity and subsequently inhibiting BCAA catabolism.^[^ ^32^, ^33^ ^]^ This phosphorylation process occurs exclusively within the mitochondria. BCKDK is crucial in the progression of various diseases through its metabolic functions, including diabetes, heart failure, and cancer.^[^ ^33^, ^34^, ^35^, ^36^ ^]^

Our previous work demonstrated that the dephosphorylation of the BCKDH complex can switch BCAA catabolism to promote liver cancer under glutamine‐deprivation conditions.^[^ ^37^ ^]^ In this study, we further discovered that BCKDK predominantly localizes in the nucleus of breast cancer cells. It acts as a modulator of homologous recombination repair (HRR) by phosphorylating RNF8, which prevents RAD51 degradation, thereby promoting tumorigenesis and resistance to DNA repair inhibitors or DNA‐damaging agents. Notably, abnormal expression of BCKDK, phosphorylated RNF8 (p‐RNF8), and RAD51 are associated with cancer progression and patient outcomes. Additionally, we screened a small molecule inhibitor, GSK180736A, which targets BCKDK's HRR function. This inhibitor shows synergistic activity when combined with clinical agents in breast cancer treatment. This finding underscores BCKDK's role in HRR, independent of its metabolic function, positioning it as a promising target for therapeutic interventions.

## 2 Results

### 2.1 Nuclear‐Localized BCKDK Enhances HRR in Breast Cancer

**Figure 1:**
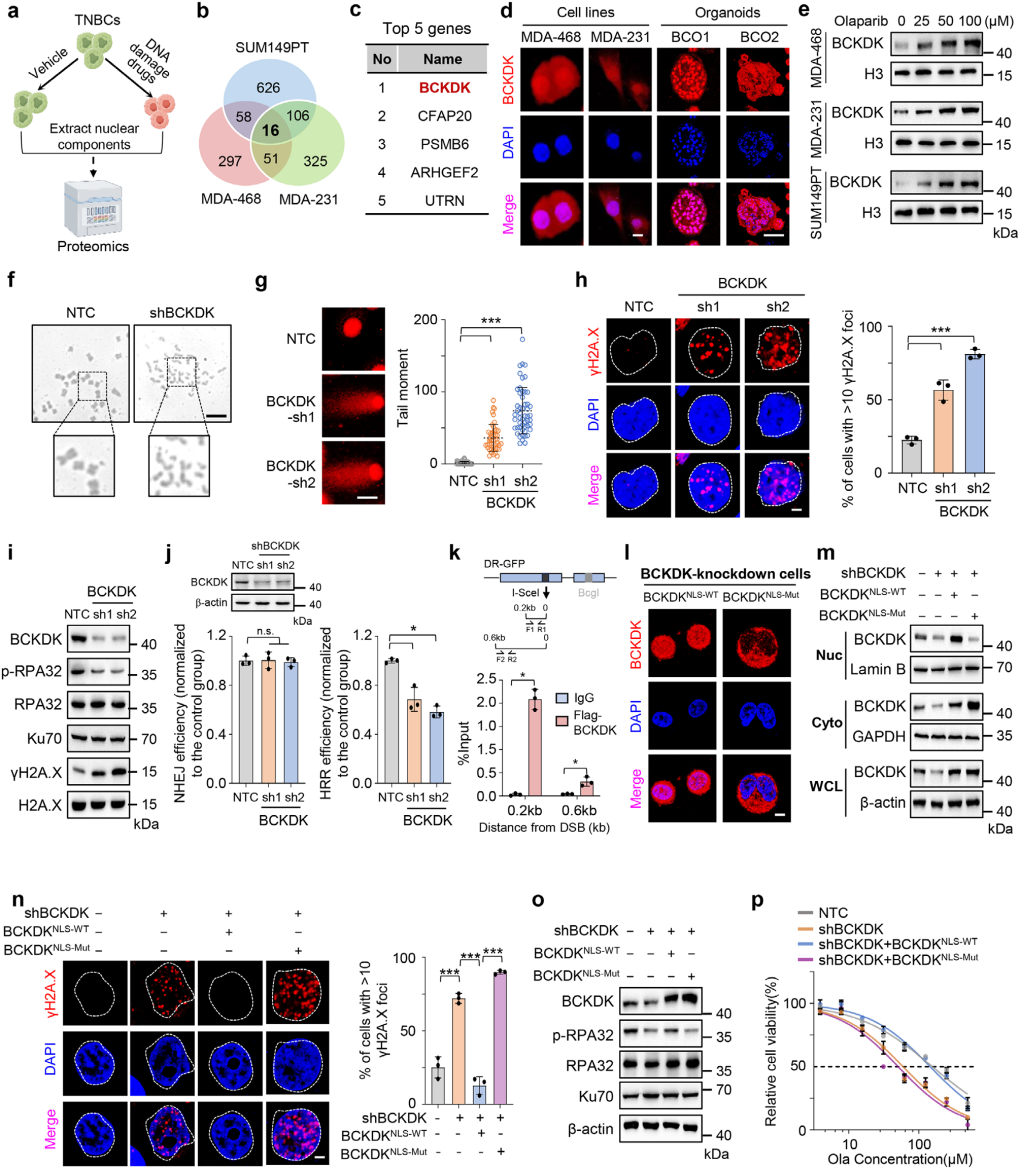
Branched‐chain α‐ketoacid dehydrogenase kinase (BCKDK) localizes in the nucleus to facilitate DNA damage repair and homologous recombination repair (HRR) in breast cancer. a) Schematic diagram for screening key genes regulating DNA damage repair. We used three triple‐negative breast cancer (TNBC) cell lines, SUM149PT, MDA‐468 and MDA‐231 in the screening experiment. Selection of these cell lines was based on their distinct genetic backgrounds and varying responses to treatment. b) Venn diagram of nuclear proteomics analysis showing the overlap of genes enriched in three groups. The red, green, and blue circles represent gene enrichment in the MDA‐468, MDA‐231, and SUM149PT cell lines, respectively. c) List of top five ranked overlapping genes in (b). d) Immunofluorescence (IF) staining of MDA‐468, and MDA‐231 cell lines and breast cancer organoids with BCKDK antibody (red) and DAPI (blue). Scale bars, 5 µm in cell lines and 10 µm in organoids (BCO1 and BCO2 were derived from patients with Luminal B breast cancer). e) Western blot analysis of BCKDK in nuclear fractions of MDA‐468, MDA‐231, and SUM149PT cells treated with Olaparib at the concentrations of 0, 25, 50, and 100 × 10^−6^ m for 48 h. f) Representative images showing increased genomic instability in MDA‐468 cells stably expressing nontargeting control vectors (NTC) or BCKDK shRNAs. Scale bar, 50 µm. g) Comet assay shows the tail moment of MDA‐468 cells stably expressing BCKDK shRNAs (sh1 and sh2) or NTC. Representative images are shown on the left, with the number of tail moment cells quantified on the right. Scale bars, 10 µm. h) γH2A.X foci (red) and DAPI (blue) visualized by IF microscopy in NTC or shBCKDK expressing MDA‐468 cells. Representative images are shown on the left, with the number of foci per cell quantified on the right. Scale bar, 5 µm. i)Western blot analysis of BCKDK, Ku70, p‐RPA32, RPA32, γH2A.X, and H2A.X levels in MDA‐468 cells stably expressing NTC or BCKDK shRNAs (sh1 and sh2). j) Analysis of *** nonhomologous end joining (NHEJ) and HRR efficiency in U2OS cells expressing NTC or BCKDK shRNAs (sh1 and sh2) by using DSB repair reporter (DRR) system. k) Chromatin immunoprecipitation (ChIP) analyses with the indicated antibodies were performed at the indicated for 48 h after I‐SceI transfection in overexpressing DR‐GFP U2OS cells. The indicated primers covering a range of distances from the cutting open site were used for the PCR. l) IF staining with an BCKDK antibody (red) and DAPI (blue) in endogenous BCKDK knockdown MDA‐468 cells, followed by transfected with BCKDK^NLS‐WT^ or BCKDK^NLS‐Mut^ vectors. Scale bar, 5 µm. m–p)MDA‐468 cells stably expressing NTC or BCKDK shRNAs were further transfected with BCKDK^NLS‐WT^, BCKDK^NLS‐Mut^, or control vectors. Each group of cells was followed by the experiments described below. Western blot analysis of BCKDK levels in the fraction of nuclear (Nuc), cytosolic (Cyto), and whole cell lysate (WCL). Lamin B, GAPDH and β‐actin were used to verify each fraction (m). IF staining of each group of cells with γH2A.X foci (red) and DAPI (blue). Representative images are shown on the left, with the number of foci per cell quantified on the right. Scale bar, 5 µm (n). Western blot analysis of cellular BCKDK, Ku70, p‐RPA32, and RPA32 levels (o). CCK8 assay determines the IC_50_ of Olaparib (Ola) treatment over 48 h (p). Western blots are representative of three independent experiments (e,i,j,m,o). Error bars denote mean ± S.D. or mean ± S.E.M. (g,h,j,n,p). Statistical analyses were performed by two‐tailed Student's t‐test (p). Statistical analyses were performed by one‐way ANOVA with Tukey's multiple comparisons test (g,h,j,n). Lamin B, GAPDH, H2A.X, and β‐actin serve as loading control in the Western blot. ^*^ *p* < 0.05, ^**^ *p* < 0.01, or ^***^ *p* < 0.001 as compared to corresponding group.

To identify the critical factors involved in DNA repair mechanisms that contribute to breast cancer cell survival and resistance to DNA damage‐inducing therapy, we conducted nuclear proteomics screening using three TNBC cell lines, SUM149PT, MDA‐468, and MDA‐231 treated with or without DNA repair inhibitor Olaparib (**Figure** **1**a). Our results revealed that the expression of 16 proteins was increased following Olaparib treatment among all three cell lines (Figure 1b; Table S1, Supporting Information), with BCKDK being the most significant (Figure 1c). Since BCKDK is known for its role in regulating the catabolic flux of BCAA that localized in mitochondria, we confirmed the nuclear localization of BCKDK by performing immunofluorescence (IF) staining, and observed that the majority of the BCKDK signal co‐localized with the nuclear counterstain DAPI in various breast cancer cell lines and patient‐derived organoids (Figure 1d; Figure S1a, Supporting Information). Western blot analysis of nuclear and cytosolic subcellular fractions also revealed that BCKDK was enriched in the nucleus (Figure S1b, Supporting Information). Meanwhile, treatment with Olaparib elevated the nuclear BCKDK in a dose‐dependent manner (Figure 1e), suggesting BCKDK functions in the nuclear regulatory events in breast cancer. Additionally, our data showed that nuclear BCKDK increased along with a decrease in the levels of cytosolic BCKDK following Olaparib treatment, while total cellular levels of BCKDK exhibited negligible changes (Figure S1c,d, Supporting Information). These data suggested that the observed increase in nuclear BCKDK upon PARP inhibitor treatment is attributed to a translocation event rather than an increase in the overall expression or stability of the protein. To study the function of nuclear BCKDK in DNA damage repair, we conducted a metaphase spread experiment and found that knockdown of BCKDK by short‐hairpin RNAs leads to chromosomal breakage (Figure 1f). Moreover, there was an increase in chromosome tailing (Figure 1g) and fluorescence intensity of γH2A.X (Figure 1h) in BCKDK‐knockdown MDA‐468 cells. These findings suggest that BCKDK plays a crucial role in the repair of DNA double‐strand breaks.

DNA double‐strand break repair primarily involves two mechanisms: HRR and nonhomologous end joining (NHEJ).^[^ ^21^, ^38^ ^]^ To further evaluate the precise function of BCKDK, we performed cell cycle synchronization experiments and found that both whole cellular and nuclear protein levels of BCKDK increased during the S and G2 phases (Figure S1e, Supporting Information). Additionally, we found that the expression of p‐RPA32 and formation of RAD51 foci, two markers of HRR, was reduced in BCKDK‐silenced breast cancer cells (Figure 1i; Figure S1f,g, Supporting Information), while the expression of the Ku70 and formation of 53BP1 foci, two markers of NHEJ, remained unaffected (Figure 1i; Figure S1f,g, Supporting Information). To further investigate the role of BCKDK in DNA damage repair, we utilized the DSB repair reporter (DRR) system to evaluate the efficiency of both HRR and NHEJ. Our findings revealed a significant reduction in HRR efficiency in BCKDK knockdown cells, while overexpression of BCKDK led to an increase in HRR efficiency (Figure 1j, Figure S1h, Supporting Information). Notably, NHEJ efficiency was not influenced by changes in BCKDK expression (Figure 1j, Figure S1h, Supporting Information). Collectively, these results indicate that BCKDK primarily regulates HRR rather than NHEJ in breast cancer cells. To assess whether BCKDK is recruited to sites of DNA damage, we performed chromatin immunoprecipitation (ChIP) assays using an I‐SceI induced HRR system. The results indicate that BCKDK is recruited to the sites of DNA damage upon induction (Figure 1k). Taken together, our results suggest that nuclear BCKDK enhances HRR by being recruited to the sites of DNA damage.

The BCKDK protein was predicted to have one nuclear localization signal (NLS) sequence, ^226^SPKKI^230^, as analyzed by cNLS Mapper (http://nls‐mapper.iab.keio.ac.jp/cgi‐bin/NLS_Mapper_form.cgi).^[^ ^39^ ^]^ To determine whether this NLS sequence controls BCKDK translocation, we introduced nonsynonymous mutations (AAAAA) to the NLS sequence of BCKDK (BCKDK^NLS‐Mut^). The results showed that forced expression of wild‐type BCKDK (BCKDK^NLS‐WT^) in endogenous BCKDK‐knockdown MDA‐468 cells restored nuclear BCKDK levels, while BCKDK^NLS‐Mut^ failed to do so (Figure 1l,m). Consistently, ectopic expression of GFP‐labeled wild‐type BCKDK (GFP‐BCKDK^NLS‐WT^) was enriched in the nucleus following Olaparib treatment, whereas a mutated version of BCKDK lacking a nuclear localization sequence (GFP‐BCKDK^NLS‐Mut^) resulted in a lack of nuclear localization both with and without PARP inhibition (Figure S1i, Supporting Information). Moreover, ectopic expression of BCKDK^NLS‐WT^, but not BCKDK^NLS‐Mut^, inhibited DNA damage (Figure 1n) and enhanced the levels of HRR marker p‐RPA32 (Figure 1o) in BCKDK‐silenced cells. As a consequence, knockdown of BCKDK heightened the sensitivity of MDA‐468 cells to Olaparib (Figure 1p). Notably, BCKDK^NLS‐WT^, but not BCKDK^NLS‐Mut^, completely reversed this sensitivity (Figure 1p). Taken together, our results suggest that nuclear localization of BCKDK facilitates DNA damage repair by enhancing HRR in breast cancer.

### 2.2 BCKDK Phosphorylates RNF8 to Modulate HRR‐Mediated DNA Repair

**Figure 2:**
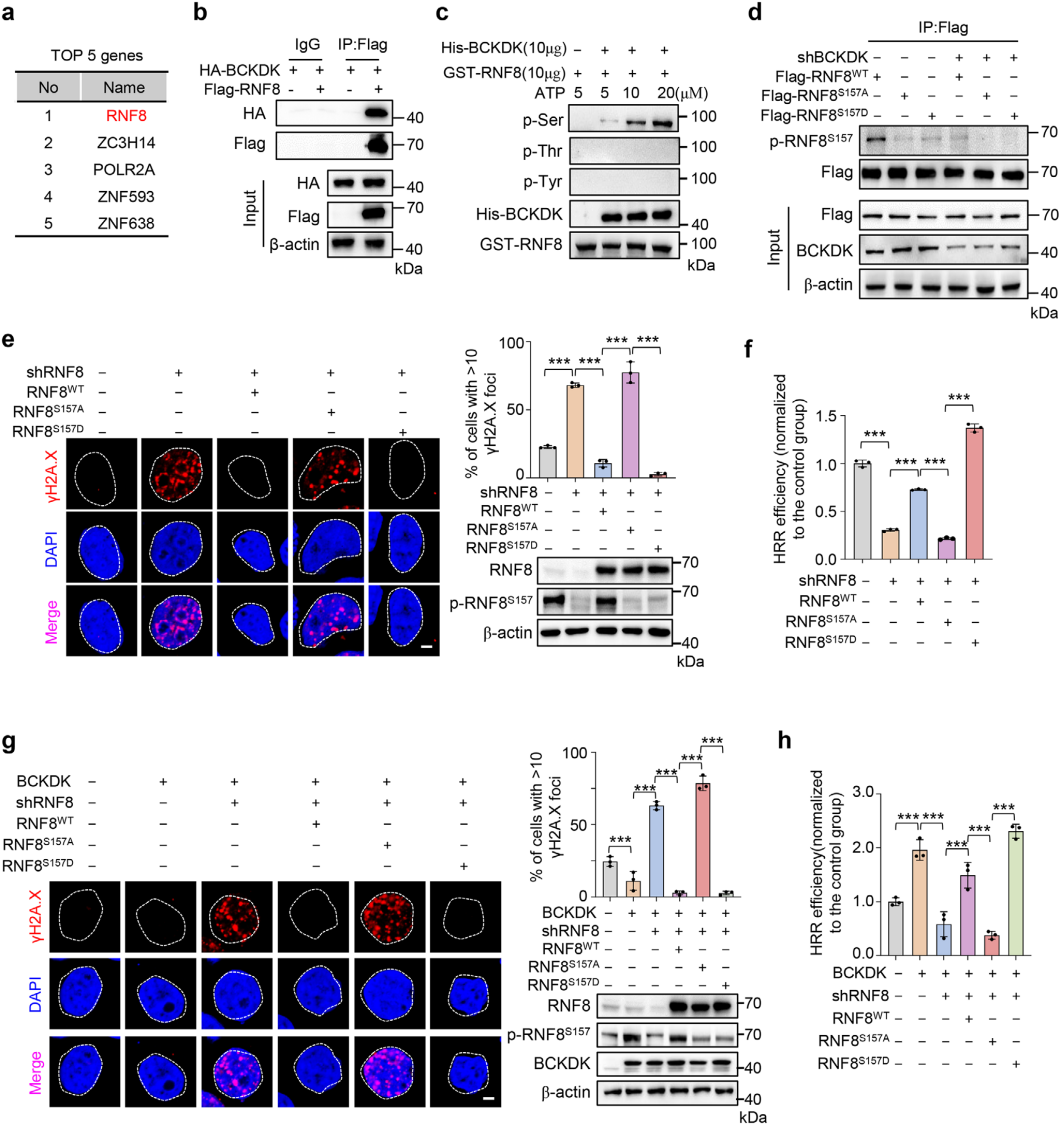
Branched‐chain α‐ketoacid dehydrogenase kinase (BCKDK) binds with and phosphorylates RNF8 at S157 to modulate homologous recombination repair (HRR). a) Immunoprecipitation‐mass spectrometry (IP‐MS) analysis of nuclear proteins interacting with BCKDK in Flag‐BCKDK overexpressed MDA‐468 cells. The top five rated DNA damage repair proteins are listed. b) Co‐IP assay in MDA‐468 cells co‐transfected with HA‐BCKDK and Flag‐tagged RNF8. Cell lysates were immunoprecipitated with Flag antibody, followed by Western blot analysis with antibodies against HA or Flag. c) In vitro kinase assay analysis of GST‐RNF8 phosphorylation in the absence or presence of His‐BCKDK by adding ATP at the indicated concentrations. d) MDA‐468 cells stably expressing nontargeting control vectors (NTC) or BCKDK shRNAs were transfected with Flag‐RNF8^WT^, Flag‐RNF8^S157A^, or Flag‐RNF8^S157D^ vectors. Cells were harvested and subjected to IP with Flag antibody, followed by Western blot to detect p‐RNF8^S157^, Flag, and BCKDK levels. e) Immunofluorescence (IF) staining with γH2A.X foci (red) and DAPI (blue) in MDA‐468 cells stably expressing NTC or RNF8 shRNAs, followed by transfected with RNF8^WT^, RNF8^S157A^, RNF8^S157D^, or control vectors. Representative images are shown on the left, with the number of foci per cell quantified on the right top, Western blot analysis of RNF8 and p‐RNF8^S157^ was shown on the right bottom. Scale bar, 5 µm. f) HRR efficiency levels were tested via I‐Scel HRR system in U2OS cells stably expressing NTC or RNF8 shRNAs, followed by transfected with RNF8^WT^, RNF8^S157A^, RNF8^S157D^, or control vectors. g) IF staining with γH2A.X foci (red) and DAPI (blue) in MDA‐468 cells stably expressing BCKDK and NTC or RNF8 shRNAs, followed by transfected with RNF8^WT^, RNF8^S157A^, RNF8^S157D^, or control vectors. Representative images are shown on the left, with the number of foci per cell quantified on the right top, Western blot analysis of RNF8, p‐RNF8^S157^, and BCKDK was shown on the right bottom. Scale bar, 5 µm. h) HRR efficiency levels were tested via I‐Scel HRR system in U2OS cells stably expressing BCKDK and NTC or RNF8 shRNAs, followed by transfected with RNF8^WT^, RNF8^S157A^, RNF8^S157D^, or control vectors. Western blots are representative of three independent experiments (b,c,d,e,g). Error bars denote mean ± S.D. or mean ± S.E.M. (e,f,g,h). Statistical analyses were performed by one‐way ANOVA with Tukey's multiple comparisons test (e,f,g,h). β‐actin serves as loading control in the Western blot. ^*^ *p* < 0.05, ^**^ *p* < 0.01, or ^***^ *p* < 0.001 as compared to corresponding group.

To elucidate the underlying mechanism by which BCKDK regulates HRR, we conducted immunoprecipitation‐mass spectrometry (IP‐MS) based proteomics on the nuclear fraction of MDA‐468 cells. Our findings indicate that E3 ubiquitin‐protein ligase RNF8 is prominently ranked among BCKDK‐associated DNA damage repair proteins (**Figure** **2**a; Table S2, Supporting Information). To confirm this result, co‐immunoprecipitation (Co‐IP) experiments showed that BCKDK overexpressed in MDA‐468 cells specifically associates with RNF8 protein (Figure 2b). A pull‐down assay using purified recombinant proteins further demonstrated the direct interaction between BCKDK and RNF8 (Figure S2a, Supporting Information), indicating that BCKDK binds to RNF8 in the nucleus.

Given that BCKDK is a protein kinase, we next tested whether RNF8 acts as a phosphorylation substrate of BCKDK. An in vitro kinase assay showed that BCKDK phosphorylates RNF8 on a serine residue, rather than threonine or tyrosine residues (Figure 2c). Moreover, IP experiments detected a reduction of the serine phosphorylation of RNF8 in BCKDK‐knockdown cells using a pan‐phospho‐serine antibody (Figure S2b, Supporting Information). Since RNF8 was predicted to have only one phosphorylation site at Serine 157 (S157) according to the online prediction tool (https://services.healthtech.dtu.dk/services/NetPhos‐3.1/),^[^ ^40^ ^]^ we introduced a mutation at S157 of RNF8 (RNF8^S157A^) and found that BCKDK was unable to phosphorylate RNF8^S157A^ compared with wild‐type RNF8 (RNF8^WT^) in vitro (Figure S2c, Supporting Information). Additionally, we developed an antibody that specifically recognizes the phosphorylation of RNF8 at S157. This antibody exclusively detects the phosphorylation of RNF8^WT^, but not the RNF8^S157A/D^ mutations (Figure 2d). Furthermore, BCKDK knockdown reduced the levels of RNF8^WT^ phosphorylation at S157 in MDA‐468 cells (Figure 2d), indicating that BCKDK phosphorylates RNF8 at the serine 157 residue.

To investigate the role of RNF8 phosphorylation at the S157 in DNA damage and HRR, we generated a phosphorylation mimic mutation (RNF8^S157D^) and a phospho‐deficient mutation (RNF8^S157A^) of RNF8. Western blot analysis revealed increased DNA damage (Figure 2e; Figure S2d,e, Supporting Information) and reduced HRR ability (Figure 2f) in endogenous RNF8 knockdown cells compared to the control group. Furthermore, ectopic expression of RNF8^S157D^, but not RNF8^S157A^, reduced DNA damage levels (Figure 2e; Figure S2d,e, Supporting Information) and enhanced HRR efficiency (Figure 2f) in RNF8‐silenced cells. These findings suggest that RNF8 modulates DNA damage repair through its phosphorylation at S157. To determine whether BCKDK regulates HRR by phosphorylating RNF8 at S157, we conducted further experiments. Our results indicated that knockdown of RNF8 impaired BCKDK‐mediated DNA damage repair (Figure 2g; Figure S2f,g, Supporting Information) and HRR (Figure 2h). Furthermore, forced expression of ectopic RNF8^S157D^, but not RNF8^S157A^, reduced DNA damage levels (Figure 2g; Figure S2f,g, Supporting Information) and restored HRR efficiency (Figure 2h). Collectively, these data demonstrate that BCKDK promotes the HRR‐mediated DNA repair process by binding to and phosphorylating RNF8 at S157.

To further investigate the protein kinase activity of BCKDK on RNF8 phosphorylation, we generated a BCKDK mutation (BCKDK^R174G^) that impairs its known catalytic domain responsible for phosphorylating the BCAA catabolic enzyme BCKDHA.^[^ ^41^ ^]^ Western blot analysis showed that knockdown of BCKDK reduced phosphorylated RNF8 and BCKDHA levels, while ectopic expression of wild‐type BCKDK rescued phosphorylation levels of both RNF8 and BCKDHA (Figure S2h, Supporting Information). Surprisingly, although BCKDK^R174G^ failed to restore BCKDHA phosphorylation, it had no effect on RNF8 phosphorylation in BCKDK‐knockdown cells (Figure S2h, Supporting Information). Consistently, ectopic expression of BCKDK^R174G^ still decreased DNA damage (Figure S2i, Supporting Information) and promoted HRR (Figure S2j, Supporting Information). These findings suggest that nuclear BCKDK may utilize an alternative kinase domain to phosphorylate RNF8, and its effects on DNA damage repair are independent of BCKDHA‐mediated BCAA catabolism.

### 2.3 BCKDK‐Mediated RNF8 Phosphorylation Stabilizes RAD51 Protein

It has been reported that RNF8 promotes the repair of DNA double‐strand breaks by facilitating the ubiquitination of histones, such as H2A.X and H3.^[^ ^42^, ^43^ ^]^ However, we found that knockdown of BCKDK did not change the ubiquitination of H2A.X (Figure S3a, Supporting Information). Moreover, although ectopic expression of RNF8^WT^ restored H2A.X ubiquitination levels in RNF8‐silenced cells, the phospho‐deficient mutation RNF8^S157A^ still exhibited this ability (Figure S3b, Supporting Information). To investigate why RNF8 S157 phosphorylation is not involved in the ubiquitination of H2A.X, we utilized AlphaFold 3 and PyMOL to study the interaction between RNF8 and H2A.X. Our results indicate that the binding site for H2A.X on RNF8 is distanced from S157 (Figure S3c, Supporting Information). Additionally, our results showed that S157 phosphorylation has no impact on H2A.X binding (Figure S3d, Supporting Information). These data suggest that RNF8 S157 phosphorylation is not involved in RNF8's binding with H2A.X and its ubiquitin modification of H2A.X, and histone ubiquitination may not be involved in BCKDK‐mediated DNA damage repair.

**Figure 3:**
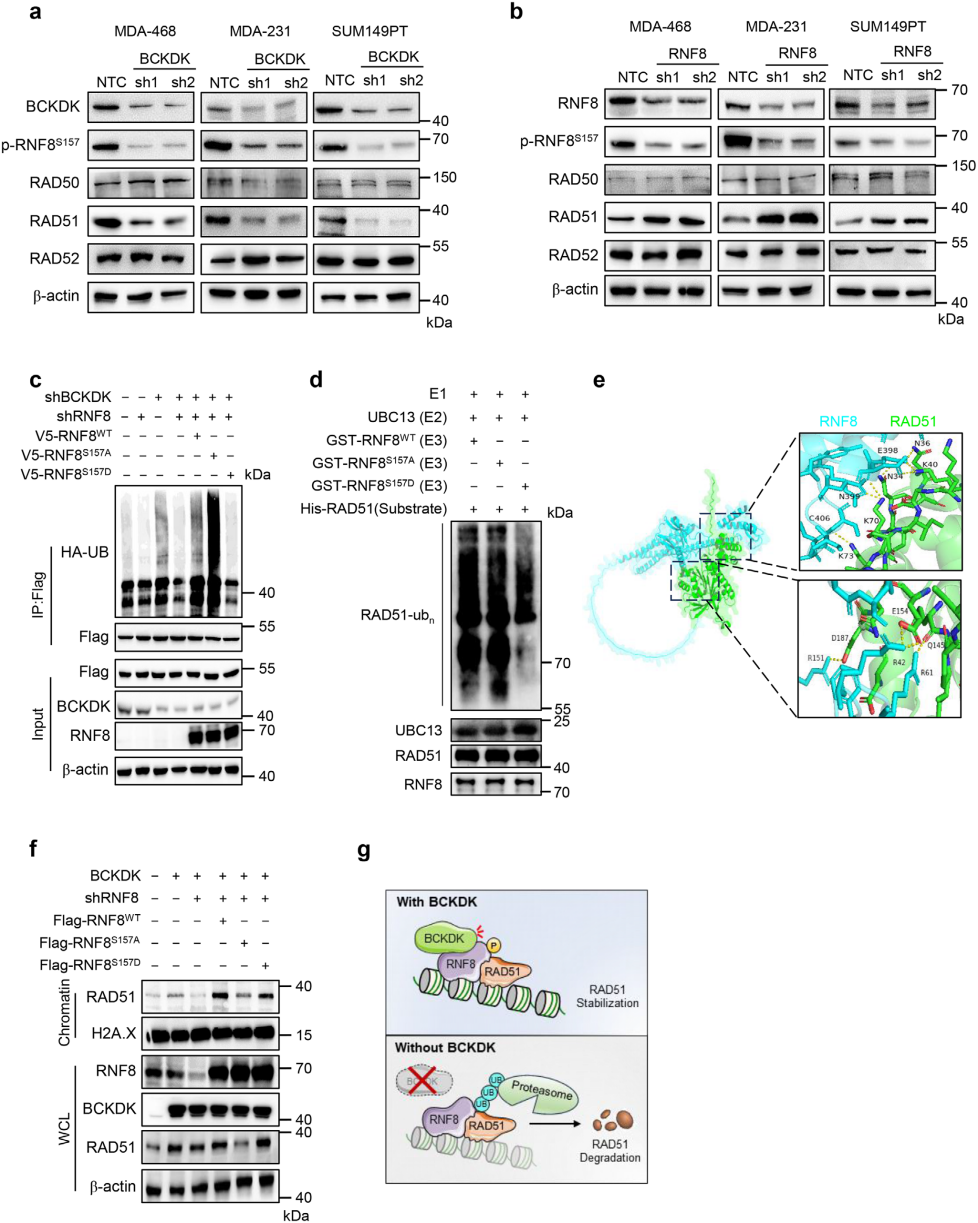
Branched‐chain α‐ketoacid dehydrogenase kinase (BCKDK) phosphorylates RNF8 at S157 to prevent ubiquitin‐mediated RAD51 degradation. a) Western blot analysis of BCKDK, p‐RNF8^S157^, RAD50, RAD51, and RAD52 levels in MDA‐468, MDA‐231, and SUM149PT cells transfected with nontargeting control vectors (NTC) or shBCKDK (sh1 or sh2). b) Western blot analysis of RNF8, p‐RNF8^S157^, RAD50, RAD51, and RAD52 levels in MDA‐468 MDA‐231, and SUM149PT cells transfected with NTC or shRNF8 (sh1 or sh2). c) Flag‐RAD51 and HA‐tagged ubiquitin (HA‐Ub) expressing HEK293T cells transfect with BCKDK shRNAs, RNF8 shRNAs, V5‐RNF8^WT^, V5‐RNF8^S157A^, V5‐RNF8^S157D^, or control vectors for 48 h, followed by treating with 10 × 10^−6^ m MG132 for 8 h before collection. Immunoprecipitation was performed by using anti‐Flag antibody or IgG. Polyubiquitination of Flag‐RAD51 were detected by Western blot. d) In vitro ubiquitination assay, substrate (RAD51) was incubated with ubiquitin activating enzyme (E1 in kit), UBC13 (E2) and GST‐RNF8^WT^, GST‐RNF8^S157A^ or GST‐RNF8^S157D^ (E3) along with Mg^2+^ and ATP. e) Diagram illustrating the amino acid residues of RAD51 bound to RNF8 by Alphafold 3 and Pymol. Green ribbon representation of the RAD51, blue ribbon representation of RNF8. f) Western blot analysis of RNF8, BCKDK, and RAD51 proteins in chromatin fractions or whole cell lysis (WCL) of MDA‐468 cells stably expressing BCKDK and NTC or RNF8 shRNAs, followed by transfected with Flag‐RNF8^WT^, Flag‐RNF8^S157A^, Flag‐RNF8^S157D^, or control vectors. g) A schematic illustrating how BCKDK binds to and phosphorylates RNF8, which in turn protects RAD51 from ubiquitin‐mediated proteasomal degradation on the chromatin. Western blots are representative of three independent experiments (a,b,c,d,f). β‐actin and H2A.X serve as loading control in the Western blot.

Therefore, we aimed to elucidate the role of RNF8 in the regulation of BCKDK‐mediated DNA damage repair. By examining DNA damage repair‐associated factors, we observed a large decrease in RAD51 protein levels following BCKDK knockdown (**Figure** **3**a), while RAD51 levels increased upon RNF8 knockdown in breast cancer cell lines (Figure 3b). In contrast, RAD50 and RAD52 protein levels remained unchanged (Figure 3a,b). Since *RAD51* mRNA levels were unaffected by both BCKDK and RNF8 knockdown (Figure S3e, Supporting Information), we hypothesized that BCKDK and RNF8 regulated RAD51 expression via a ubiquitin‐mediated protein degradation pathway. Supporting this hypothesis, RAD51 protein levels were not altered by BCKDK knockdown when treated with MG132, a proteasome inhibitor (Figure S3f, Supporting Information). Western blot analysis revealed that RAD51 ubiquitination levels were increased in BCKDK‐silenced breast cancer cells, an effect that was restored with RNF8 knockdown (Figure 3c). Furthermore, ectopic expression of RNF8 mutants (RNF8S^157A/D^) in BCKDK and RNF8 double knockdown cells showed that only the phospho‐deficient mutation RNF8^S157A^ enhanced RAD51 ubiquitination, while the phosphorylation mimic mutation RNF8^S157D^ did not alter RAD51 ubiquitination (Figure 3c). In addition, in vitro ubiquitination assays confirmed that RNF8 S157 phosphorylation regulates RAD51 ubiquitination (Figure 3d). AlphaFold 3 and PyMOL analysis showed that the binding site for RAD51 on RNF8, particularly R151, is located near the S157 phosphorylation site (Figure 3e). Co‐IP assays further revealed that the RNF8 S157 phosphorylation is involved in its interaction with RAD51 (Figure S3g, Supporting Information). These findings suggest that BCKDK inhibits RAD51 ubiquitination by phosphorylating RNF8 at S157, and this phosphorylation may modulate the interaction between RNF8 and RAD51, thereby regulating RAD51's ubiquitination.

When DNA damage occurs, RNF8 accumulates on chromosomes and recruits DNA damage repair proteins to facilitate the repair of DNA strand breaks.^[^ ^43^ ^]^ We extracted chromosome components and found that overexpression of BCKDK stabilized RAD51 and increased its accumulation on chromosomes (Figure 3f). Moreover, forced expression of RNF8^S157A^ inhibited chromosomal aggregation of RAD51, while expression of RNF8S^157D^ promoted RAD51 accumulation on chromosomes in BCKDK‐overexpressing and endogenous RNF8‐silenced MAD‐468 cells (Figure 3f). Collectively, our results suggest a working model in which BCKDK binds to and phosphorylates RNF8 to recruit and stabilize RAD51 on chromosomes. In the absence of BCKDK, dephosphorylated RNF8 can stimulate RAD51 ubiquitination, thereby accelerating the proteasomal degradation of RAD51 (Figure 3g).

### 2.4 BCKDK/p‐RNF8/RAD51 Axis Promotes Cell Resistance Against DNA Damage‐Inducing Therapy

**Figure 4:**
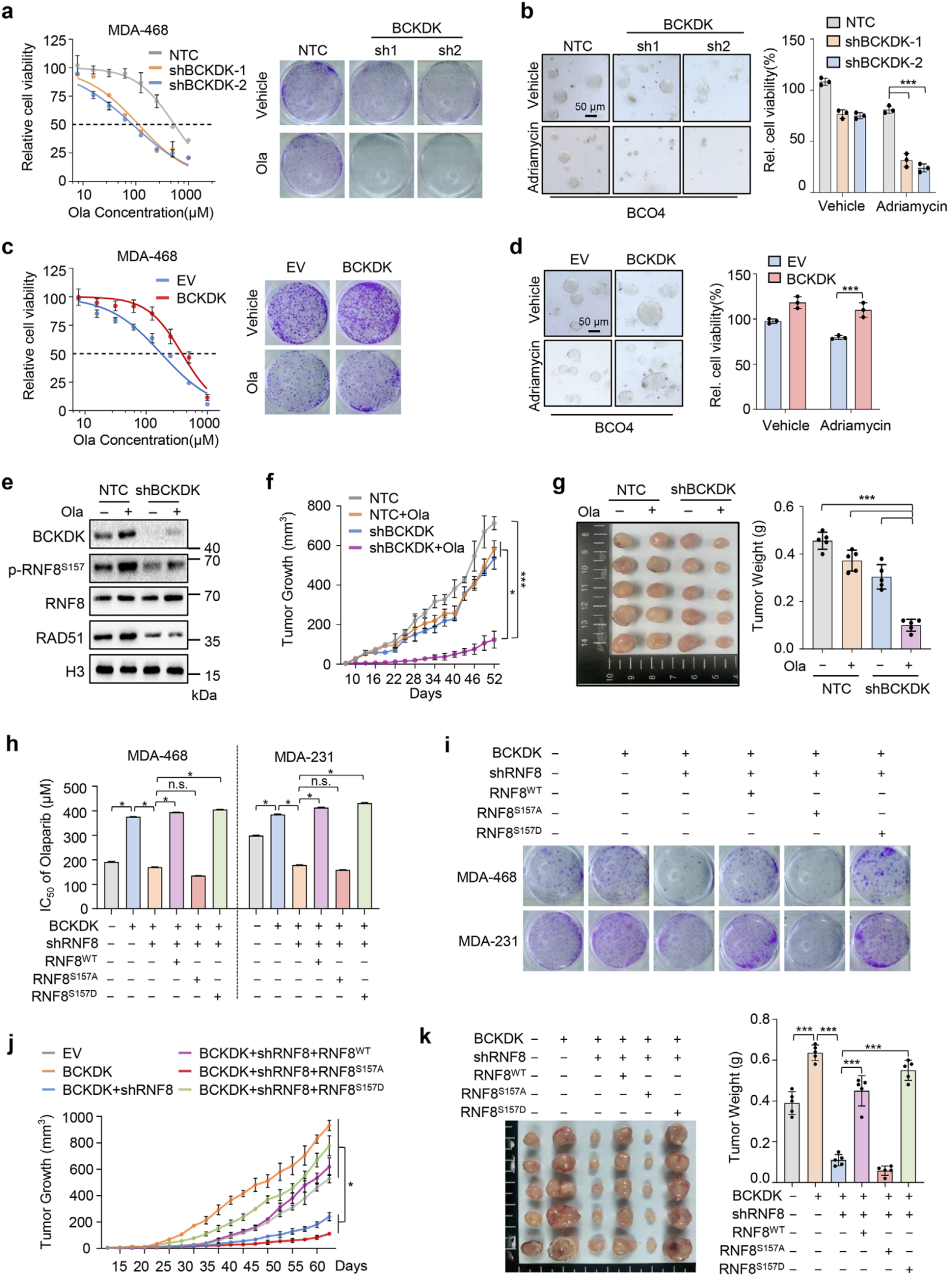
The branched‐chain α‐ketoacid dehydrogenase kinase (BCKDK) mediated RNF8 phosphorylation contributes to resistance against DNA damage‐inducing drugs in breast cancer. a) The IC_50_ values of Olaparib (Ola) treatment over 48 h (left), and colony formation with or without 1 × 10^−6^ m Olaparib treatment for 14 days (right) were examined in MDA‐468 cells stably expressing nontargeting control vectors (NTC) or shBCKDK (sh1, sh2). b) Breast cancer organoid BCO4 stably expressing NTC or BCKDK shRNAs (sh1 and sh2) were treated with or without DNA damage agent 0.1 × 10^−6^ m Adriamycin for 6 days. Representative micrographs were shown (left) and relative cell viability were analyzed (right). Scale bar, 50 µm. c) The IC_50_ values of Olaparib (Ola) treatment over 48 h (left), and colony formation with or without 1 × 10^−6^ m Olaparib treatment for 14 days (right) were determined in MDA‐468 cells stably expressing EV or BCKDK. d) Breast cancer organoids (BCO4) stably expressing EV or BCKDK were treated with or without 0.1 × 10^−6^ m Adriamycin for 6 days. Representative micrographs were shown (left) and relative cell viability were analyzed (right). Scale bar, 50 µm. e) Nuclear Western blotting analysis of BCKDK, RNF8, p‐RNF8^S157^, and RAD51 levels in Olaparib resistant MDA‐468 cells stably expressing NTC or BCKDK shRNAs. f,g) MDA‐468 cells stably expressing NTC or BCKDK shRNAs were subcutaneously injected in BALB/c nude mice following treatment with 25 mg kg^−1^ Olaparib or vehicle (*n* = 5 per group). Tumor growth (f), tumor images (g, left), and tumor mass (g, right) were measured at the experiment. h,i) MDA‐468 or MDA‐231 cells stably expressing BCKDK or RNF8 shRNAs were transfected with Flag‐RNF8^WT^, Flag‐RNF8^S157A^, Flag‐RNF8^S157D^, or control vectors, IC_50_ of Olaparib (Ola) treatment over 48 h (h) and colony formation with or without 1 × 10^−6^ m Olaparib treatment for 14 days (i) were determined. j,k) BCKDK overexpression and RNF8 knockdown MDA‐468 cells transfected with Flag‐RNF8^WT^, Flag‐RNF8^S157A^, Flag‐RNF8^S157D^, or control vectors were subcutaneously injected in BALB/c nude mice, following treatment with 25 mg kg^−1^ Olaparib or vehicle (*n* = 5 per group). Tumor growth (j), tumor images (k, left), and tumor mass (k, right) were measured at the experiment. Western blots are representative of three independent experiments (e). Error bars denote mean ± S.D. or mean ± S.E.M. (a,b,c,d,f,g,h,j,k). Statistical analyses were performed by two‐tailed Student's *t*‐test (a,c), one‐way or two‐way ANOVA with Tukey's multiple comparisons test (b,d,f,g,h,j,k). β‐actin and H3 serves as loading control in the Western blot. ^*^ *p* < 0.05, ^**^ *p* < 0.01, or ^***^ *p* < 0.001 as compared to corresponding group. n.s., not significant.

To explore the therapeutic potential of targeting the BCKDK‐mediate HRR, we investigated its impact on tumor response to DNA damage‐inducing therapies. Notably, knockdown of BCKDK sensitized MDA‐468, MDA‐231, and SUM149PT cells to Olaparib, as evidenced by both IC_50_ measurements for cell viability experiments and colony formation assays (**Figure** **4**a; Figure S4a,e, Supporting Information). An increase in sensitivity to Adriamycin was also observed in BCKDK knockdown organoids derived from breast cancer patients (Figure 4b; Figure S4b,e, Supporting Information). Additionally, overexpression of BCKDK counteracted the anti‐tumor effects of Olaparib or Adriamycin in the breast cancer lines (Figure 4c; Figure S4c,f, Supporting Information) or organoids (Figure 4d; Figure S4d,f, Supporting Information), respectively. Moreover, treatment with Olaparib increased RNF8 phosphorylation and RAD51 expression, which was diminished by BCKDK knockdown (Figure 4e; Figure S4g, Supporting Information). These findings indicate that elevated BCKDK expression contributes to resistance against DNA damage‐inducing drugs in breast cancer.

To further investigate the in vivo effect of BCKDK on tumor growth in response to DNA damage, xenograft tumor growth experiments using MDA‐468 cells were conducted in nude mice. We found that BCKDK knockdown dramatically reduced tumor growth rate and tumor mass under Olaparib treatment, while Olaparib had no effect on control tumors (Figure 4f,g). Consistent with in vitro results, RNF8 phosphorylation and RAD51 protein levels were increased in Olaparib‐treated tumor samples and were rescued by BCKDK knockdown (Figure S4h, Supporting Information). These results demonstrate that targeting BCKDK sensitizes breast cancer to the DNA repair inhibitor in vivo.

We next examined the role of the BCKDK/p‐RNF8/RAD51 axis in contributing to therapy resistance in breast cancer. Upon treatment with Olaparib, we found that overexpression of BCKDK increased resistance to Olaparib. This effect was reversed by knocking down RNF8 in MDA‐468 or MDA‐231 cells, as demonstrated by IC_50_ assays and colony formation assays (Figure 4h,i). Moreover, forced expression of RNF8^WT^ or RNF8^S157D^, but not RNF8^S157A^, promoted cell viability and colony formation in BCKDK‐overexpressing and endogenous RNF8‐silenced breast cancer cells (Figure 4h,i). Furthermore, analysis of mouse xenografts by implanting the above groups of MDA‐468 cells showed that BCKDK enhanced tumor volume and mass compared to the control group upon Olaparib treatment, with a restoration observed in RNF8‐silenced tumors (Figure 4j,k). BCKDK‐overexpressing and RNF8‐silenced MDA‐468 tumors expressing RNF8^WT^ or RNF8^S157D^, but not RNF8^S157A^, could restore tumor volume and mass, under Olaparib treatment (Figure 4j,k). This indicates that the BCKDK/p‐RNF8/RAD51 axis may contribute to HRR for the DNA repair inhibitor resistance both in vitro and in vivo.

Taken together, these findings demonstrate that BCKDK‐mediated RNF8 phosphorylation at S157 plays a critical role in tumor progression and contributes to resistance against DNA damage‐inducing therapy in breast cancer.

### 2.5 Aberrant Nuclear BCKDK Expression Predicts Prognosis of Breast Cancer Patients

**Figure 5:**
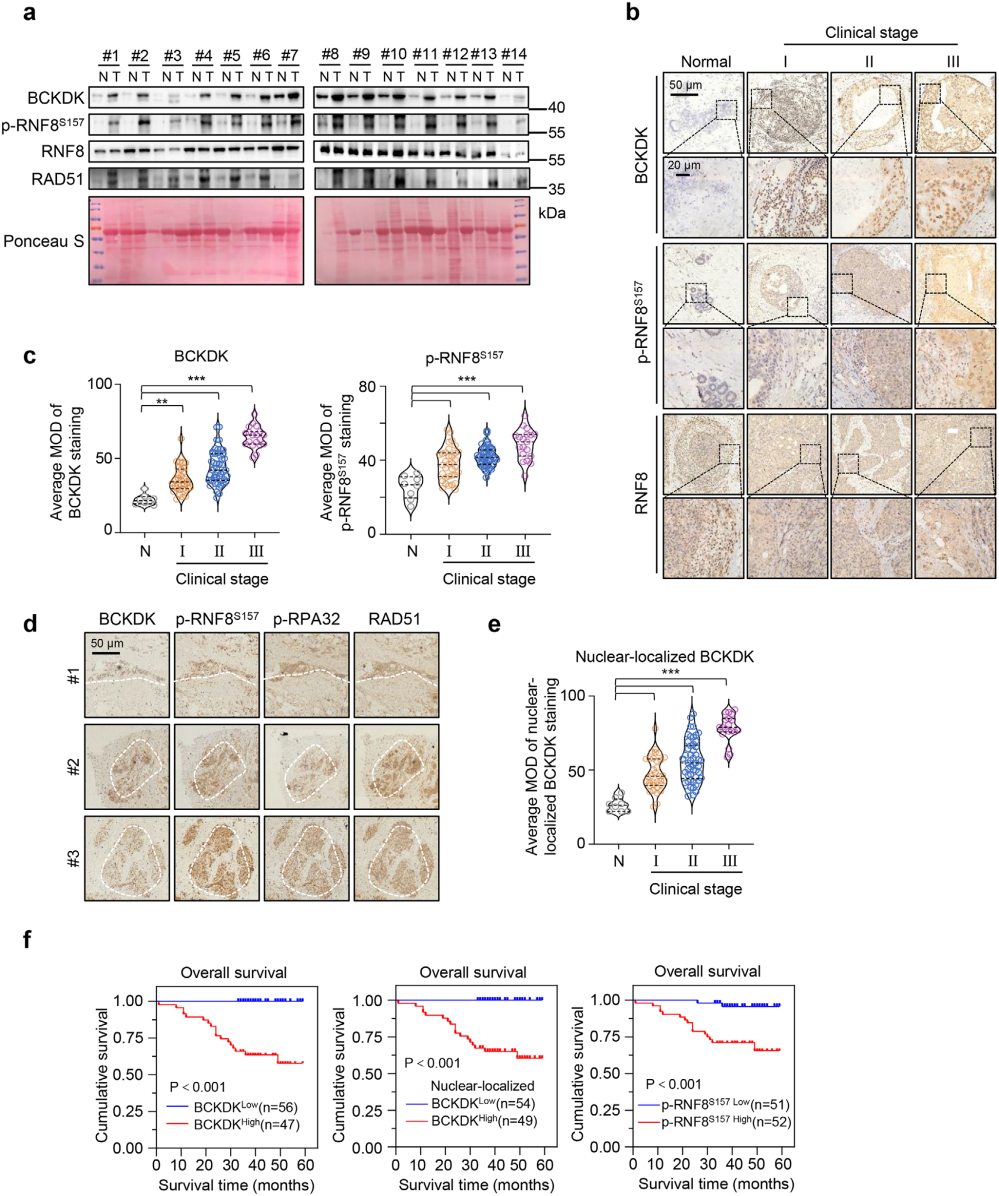
Aberrant branched‐chain α‐ketoacid dehydrogenase kinase (BCKDK)/p‐RNF8^S157^/RNF8 axis predicts breast cancer progression and mortality. a) Western blot analysis of BCKDK, p‐RNF8^S157^, RNF8, and RAD51 levels in 14 pairs of clinically matched with tumor‐adjacent noncancerous tissues (N) and breast cancer tissues (T). b) Representative immunohistochemistry (IHC) images showing BCKDK, p‐RNF8^S157^, or RNF8 levels in normal breast tissue (normal) and breast cancer specimens of different clinical stages (I‐III). Scale bars, 50 µm. Insets, fourfold magnification; scale bars, 20 µm. c) Statistical quantification of the mean intensity of BCKDK (c, left), and p‐RNF8^S157^(c, right) staining using HistoQuest software (healthy donors (*N*), *n* = 8; patients with breast cancer, stage I (*n* = 34), II (*n* = 50), and III (*n* = 19)). d) Representative IHC images of BCKDK, p‐RNF8^S157^, p‐RPA32, or RAD51 staining in the consecutive sections from breast cancer patients. Scale bars, 50 µm. e) Statistical quantification of the mean intensity of nuclear‐localized BCKDK staining using HistoQuest software (healthy donors (*N*), *n* = 8; patients with breast cancer, stage I (*n* = 34), II (*n* = 50), and III (*n* = 19)). f) Univariate Kaplan–Meier analysis of patients with low versus high levels of BCKDK, nuclear‐localized BCKDK, and p‐RNF8^S157^. Western blots are representative of three independent experiments (a). Error bars denote mean ± S.D. or mean ± S.E.M. c,e). Statistical analyses were performed by one‐way ANOVA with Tukey's multiple comparisons test (c,e) or log‐rank test (f). Ponceau S served as a loading control. ^*^ *p* < 0.05, ^**^ *p* < 0.01, or ^***^ *p* < 0.001 as compared to corresponding group.

To evaluate the potential clinical significance of the BCKDK‐mediated regulatory pathway in patient samples, we first assessed the levels of BCKDK, p‐RNF8^S157^, and RAD51 in 14 paired breast cancer lesions and adjacent noncancerous tissue samples. Western blot analysis showed increased levels of BCKDK, p‐RNF8^S157^, and RAD51 proteins in tumor tissues compared to their adjacent normal tissues (**Figure** **5**a), suggesting enhanced activity of the BCKDK/p‐RNF8/RAD51 regulatory pathway in clinical tumor tissues.

Next, immunohistochemistry (IHC) was employed to examine BCKDK and p‐RNF8^S157^ expression in a retrospective cohort of 103 breast cancer patients with well‐characterized clinicopathological features. This cohort comprised 34 stage I (33.01%), 50 stage II (48.54%), and 19 stage III (18.45%) breast cancer patients, classified according to tumor, node, metastasis staging (TNM).^[^ ^44^ ^]^ IHC staining results revealed that BCKDK and p‐RNF8^S157^ were weakly presented in adjacent normal tissues but highly abundant in cancer tissues (Figure 5b). Quantitative analysis of IHC data showed that both BCKDK and p‐RNF8^S157^ levels were significantly higher in clinical stage I–III primary tumors compared to normal compartments (Figure 5c). Moreover, the correlations among BCKDK, p‐RNF8^S157^, RAD51, and HRR marker p‐RPA32 were validated by IHC staining of patient samples (Figure 5d), suggesting that high BCKDK levels in tumor regions exhibit increased DNA repair ability. Notably, since the importance of BCKDK translocation to the nucleus was proven (Figure 1), we closely examined nuclear‐localized BCKDK in these clinical patient samples and found a large proportion of tumor samples exhibited strong nuclear BCKDK staining in contrast to adjacent normal tissues (Figure 5e), further demonstrating that nuclear‐localized BCKDK is associated with breast cancer progression.

Furthermore, Spearman correlation analysis revealed relationships between BCKDK/p‐RNF8^S157^ and various clinicopathological characteristics, including survival time, vital status, clinical stage, and tumor size. This indicates a strong association of BCKDK and p‐RNF8^S157^ levels with clinical staging and patient survival in breast cancer (Table S3, Supporting Information). Kaplan–Meier analysis showed that breast cancer patients with low levels of BCKDK and p‐RNF8^S157^ survived significantly longer than those with higher levels of these markers (Figure 5f). However, quantitative analysis of IHC data showed that RNF8 levels was not significantly correlated with patient tumor stage and prognosis (Figure S5a,b, Supporting Information). These results demonstrate that the BCKDK/p‐RNF8^S157^/RAD51 axis is correlated with breast cancer progression and can predict clinical prognosis.

### 2.6 Suppression of BCKDK Overcomes Resistance to DNA Damage‐Inducing Therapy in Breast Cancer

**Figure 6:**
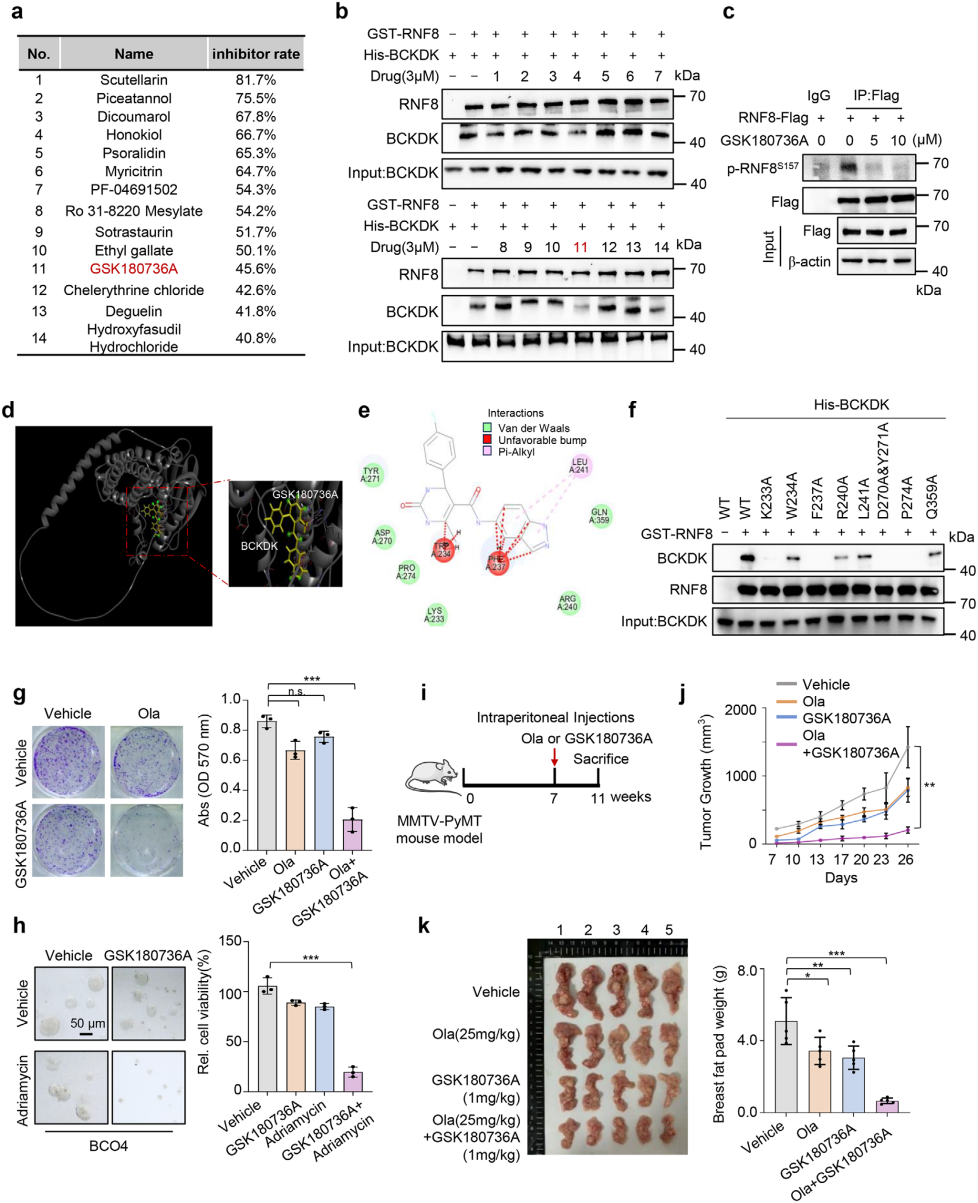
Branched‐chain α‐ketoacid dehydrogenase kinase (BCKDK) inhibition by a selective inhibitor sensitizes breast cancer cells to DNA damage‐inducing therapy. a) GST‐RNF8 and His‐BCKDK were treated with 30 × 10^−6^ m 72 kinase inhibitors in *vitro* and kinase reaction inhibition efficiency was measured by the ADP‐Glo Max assay. Top 14 kinase inhibitors were listed. b) Pull‐down assay of His‐BCKDK by GST‐RNF8 using proteins purified in *E. coli*, following by treatment with kinase inhibitors shown in (a). c) MDA‐468 cells stably expressing Flag‐RNF8 were treated with or without GSK180736A at the indicated concentrations for 24 h, cells were harvested and subjected to immunoprecipitation with Flag antibody, followed by Western blot to detect p‐RNF8^S157^ and Flag‐ RNF8 levels. d) Diagram illustrating GSK180736A bound to BCKDK. The structures of BCKDK and GSK180736A were used for the modeling using Discovery Studio. Gray ribbon representation of the BCKDK, yellow stick representation of GSK180736A. e) Two‐dimensional (2D) diagram showed the amino acid residues of BCKDK bound to GSK180736A, different colors indicated different intermolecular interactions. f) GST pull down of GST‐RNF8 by His‐BCKDK‐WT, ‐K233A, ‐W234A, ‐F237A, ‐R240A, ‐L241A, ‐D270A&Y271A, ‐P274A, or ‐Q359A was performed using *E. coli* purified proteins. g) Colony formation assay was performed in MDA‐468 cells treated with GSK180736A (4 × 10^−6^ m), Olaparib (Ola, 1 × 10^−6^ m), or a combination of both drugs for 14 days. Crystal violet staining (left) and quantitative analysis of the parallel dishes evaluated (right) on 6 days. h) Cell viability was assayed in breast cancer organoids (BCO4) treated with GSK180736A (4 × 10^−6^ m), Adriamycin (0.1 × 10^−6^ m), or a combination of both drugs for 6 days. Representative micrographs were shown (left) and relative cell viability were analyzed (right). i–k) Intraperitoneal injections of GSK180736A (1 mg kg^−1^ every 3 days), Olaparib (25 mg kg^−1^ every 3 days), or a combination of both drugs were administered to the mice of MMTV‐PyMT TNBC model. All the mice were sacrificed at 11 weeks. A schematic diagram is shown (i). Tumor growth rates were recorded after 7 days of drug administration (j). Representative images of tumors (k, left) and the quantification of breast fat pad weight (k, right) were measured at the end of the experiment. Western blots are representative of three independent experiments (b,c,f). Error bars denote mean ± S.D. or mean ± S.E.M. (a,g,h,j,k). Statistical analyses were performed by one‐way ANOVA with Tukey's multiple comparisons test (a,g,h,j,k). β‐actin served as a loading control. ^*^ *p* < 0.05, ^**^ *p* < 0.01, or ^***^ *p* < 0.001 as compared to corresponding group.

Shutting down BCKDK and its downstream pathway contributes to resistance against therapies such as Olaparib or Adriamycin (Figure 4). Therefore, we hypothesized that strategies to inhibit BCKDK's function might synergize with DNA damage‐inducing drugs. BT2 is a classic inhibitor of BCKDK, binding to BCKDK to dissociate it from the BCKDH complex and block BCKDHA phosphorylation.^[^ ^33^, ^45^ ^]^ Given that nuclear BCKDK may use an alternative kinase domain to phosphorylate RNF8 (Figure S2h–j, Supporting Information), we aim to identify inhibitors that specifically target BCKDK's function in HRR. Since BCKDK is a member of the PDK family,^[^ ^46^, ^47^ ^]^ we performed a kinase inhibition library screen targeting this family, comprising 72 small molecule inhibitors, to evaluate kinase inhibition efficiency using the ADP‐Glo Max assay. The screening identified 14 small‐molecule inhibitors that effectively inhibited BCKDK‐mediated phosphorylation of RNF8 in vitro, with an inhibition rate exceeding 40% (**Figure** **6**a). Among these, an in vitro pull‐down assay revealed that one inhibitor, GSK180736A (No.11), observably reduced the interaction between BCKDK and RNF8 (Figure 6b). Notably, GSK180736A was shown to decrease RNF8 phosphorylation at S157 in MDA‐468 cells (Figure 6c).

To confirm that GSK180736A inhibits RNF8 phosphorylation by disrupting the binding of BCKDK to RNF8, we utilized Discovery Studio to predict critical amino acid residues within BCKDK that may mediate its interaction with the drug. Our analysis identified several residues likely important for this interaction, which probably occurs through various intermolecular forces, including Van der Waals interactions (Figure 6d,e). Notably, these amino acids are located predominantly in the C‐terminal region of BCKDK. Subsequently, pull‐down assay revealed that mutations at K233, F237, D270, Y271, and P274 led to a significant reduction in the interaction between BCKDK and RNF8 (Figure 6f), suggesting these residues on BCKDK are important for its interaction with RNF8. Finally, we employed AlphaFold 3 and PyMOL to predict the binding of BCKDK with RNF8 (Figure S6a, Supporting Information). The results suggested that RNF8 binds to the C‐terminal region of BCKDK, with the binding sites (E243 and K245) located near the interaction sites (K233, W234, F237, R240, and L241) for GSK180736A. Take together, these data suggest that GSK180736A may occupy the interaction sites between BCKDK and RNF8, thereby disrupting their interaction and inhibiting RNF8 phosphorylation.

To examine the role of GSK180736A in targeting the BCKDK/p‐RNF8/RAD51 axis, we demonstrated that GSK180736A increased RAD51 ubiquitination (Figure S6b, Supporting Information). Additionally, it reduced the accumulation of both BCKDK and RAD51 on chromosomes and enhanced γH2A.X expression, without affecting the phosphorylation of BCKDHA (Figure S6c, Supporting Information). Notably, MDA‐468 cells were more sensitive to GSK180736A compared to BT2 treatment (Figure S6d, Supporting Information). These findings suggest that GSK180736A has strong specificity in targeting the BCKDK/p‐RNF8/RAD51 axis and HRR‐mediated DNA repair process in breast cancer.

Next, we investigated whether GSK180736A could reverse resistance to DNA damage‐inducing drugs. Our results showed that treatment with Olaparib or GSK180736A alone had a mild effect, while the combination of these two compounds produces a strong synergistic effect in suppressing breast cancer cell growth (Figure 6g). Additionally, GSK180736A increased the sensitivity of patient‐derived breast cancer organoids to Adriamycin across all four subtypes (Figure 6h; Figure S6e,f, Supporting Information). These findings suggest that the BCKDK inhibitor GSK180736A exhibits an in vitro anti‐tumor effect when used in combination with DNA repair inhibitor or DNA‐damaging agent. To further assess the synergistic effect in vivo, we utilized an MMTV‐PyMT mouse TNBC model,^[^ ^48^, ^49^ ^]^ which showed an enhanced BCKDK/p‐RNF8/RAD51 axis in tumor tissues compared to normal compartments (Figure S6g, Supporting Information). After tumor induction, intraperitoneal injections of GSK180736A, Olaparib, or both were started at 7 weeks (Figure 6i). In line with the in vitro data, monotherapy with GSK180736A or Olaparib provided only modest tumor inhibition (Figure 6j,k; Figure S6h,i, Supporting Information). In contrast, combination treatment resulted in significant suppression of tumor growth rate (Figure 6j), breast fat pad mass (Figure 6k), and tumor numbers (Figure S6i, Supporting Information) in the mouse breast cancer model. Collectively, these results demonstrate that the specific BCKDK inhibitor GSK180736A exhibits a strong synergistic effect with DNA damage‐inducing drugs in suppressing breast cancer.

## 3 Discussion

Under DNA replication stress conditions, DNA damage repair is crucial for the survival of tumor cells. This study reveals that BCKDK can localize within the nuclei of breast cancer cells, where it binds to and phosphorylates RNF8, thereby inhibiting the ubiquitin‐mediated degradation of RAD51. This process ultimately enhances HRR in tumor cells, promoting tumor progression and resistance to DNA damage‐inducing therapy, positioning BCKDK as a potential therapeutic target in clinical breast cancer treatment (Figure S7, Supporting Information).

HRR plays a pivotal role in tumor development, progression, and treatment. In the context of TNBC, BRCA1/2 serves as a critical biomarker for the efficacy of PARP inhibitor therapy. In BRCA1/2‐deficient TNBC patients, the loss of HRR enhances the clinical therapeutic outcomes of PARP inhibitors. However, numerous studies have demonstrated that resistance to PARP inhibitors can still occur even in the absence of BRCA1/2. This study delved into the nuclear proteomics of BRCA1/2‐deficient SUM149PT cells and BRCA1/2‐sufficient MDA‐468 and MDA‐231 (Figure 1a), revealing a high expression of BCKDK in the nucleus. Notably, overexpression of BCKDK significantly increased resistance to PARP inhibitors. This finding suggests that, in addition to BRCA1/2 deficiency, the expression and nuclear localization of BCKDK should be considered when treating TNBC patients with PARP inhibitors.

Traditionally, BCKDK is known as a key enzyme in branched‐chain amino acid catabolism, primarily located in the mitochondria. However, this research demonstrated that BCKDK can also localize in the nuclei of breast cancer cells. The nuclear presence of BCKDK is crucial for HRR and tumorigenesis, challenging traditional understanding and expanding therapeutic strategies targeting BCKDK in cancer. Additionally, we found that BCKDK can phosphorylate RNF8 (Figure 2). It is worth investigating whether the traditional BCKDK inhibitor BT2 can inhibit RNF8, as well as identifying the specific regions or sites through which BCKDK exerts its protein kinase activity. Furthermore, we discovered that phosphorylated RNF8 can inhibit the ubiquitin‐mediated degradation of RAD51 (Figure 3). This raises the question of whether RNF8 has other substrates besides RAD51. The specific relationships among BCKDK, RNF8, and RAD51, as well as the detailed process of RAD51 ubiquitination, warrant further exploration.

Our findings demonstrate that patients with high BCKDK expression exhibit resistance to DNA damage‐inducing drugs due to enhanced HRR capacity. Through small‐molecule inhibitor screening, we identified the selective BCKDK inhibitor GSK180736A. Experimental results from cell lines, organoids, and mouse models show that GSK180736A effectively enhanced the therapeutic efficacy of clinical DNA damage‐inducing drugs, including the PARP inhibitor Olaparib and DNA damage agent Adriamycin. Although GSK180736A is known as a Rho‐associated coiled‐coil kinase 1 (ROCK1) inhibitor,^[^ ^50^ ^]^ research on it remains limited and experimental. Our study broadens the potential targets of GSK180736A and provides important theoretical evidence for its clinical application in breast cancer.

## 4 Experimental Section

### Cell Culture

Human 293T, MDA‐MB‐468 (MDA‐468), MDA‐MB‐231 (MDA‐231), SUM149PT, Hs‐578T, and U2OS cells were maintained in Dulbecco's Modified Eagle's Medium (Thermo Fisher Scientific, 12 800 082). MCF7, T47D, BT474, and SKBR3 cells were cultured in RPMI‐1640 and supplemented with 1.5 g L^−1^ NaHCO_3_, 2.5 g L^−1^ glucose, and 0.11 g L^−1^ sodium pyruvate. All media was supplemented with 10% fetal bovine serum (VivaCell, Shanghai, China) and 1% penicillin‐streptomycin (HyClone, SV30010). Cell culture dishes/plates (8 001 004, 801 006, and 801 007), round coverslips and centrifuge tubes were obtained from NEST Biotechnology.

### Plasmids and Stable Cell Lines

The prepared sequences of BCKDK, RNF8, and RAD51 were inserted into the lentiviral plasmid pCDH‐3xFlag vector. shRNAs targeting BCKDK and RNF8 at the 3′UTR or CDS region were constructed in the PLKO.1 vector, and the target sequences are listed in Table S4 (Supporting Information). Briefly, targeting plasmids were co‐transfected with viral packaging plasmids (VSVG and ∆8.9) into HEK293T cells using PEI (Polysciences, 23966‐2). The collected viral supernatant and 8 µg mL^−1^ polybrene (Sigma‐Aldrich, H9268) were added to the culture medium of MDA‐468, MDA‐231, SUM149PT, MCF7, T47D, U2OS, and HEK293T cells. Infected cancer cells with 1 µg mL^−1^ puromycin (Sigma‐Aldrich, P8833) for 2 weeks were selected to establish stable cell lines.

### Colony Formation Assays

A total of 2000–5000 cells were seeded in 6‐well plates and treated them with DNA repair inhibitor (Olaparib) or DNA‐damaging agent (Adriamycin) or BCKDK inhibitor (GSK180736A) for approximately 2 weeks. The cells were fixed with ethanol for 30 min and stained them with 0.1% crystal violet solution for 30 min. After staining, the plates were washed three times with water and allowed them to air dry. Then, methanol was added to each well, and the plates were incubated with the lids on for 20 min at room temperature on a bench rocker. The optical density of each well at 570 nm (OD_570_) was measured. The average OD_570_ of the wells without cells was subtracted from the OD_570_ of each test well.

### Cell Viability Analysis

For breast cancer cell lines, cell viability was measured by using CCK8 kits, and the optical density of each well at 450 nm (OD_450_) was measured using a plate reader. For breast cancer organoids, cell viability was measured using the CellTiter‐Glo 3D reagent (Promega), and a CLARIOstar multimode microplate reader (BMG LABTECH) assessed luminescence.

### Western Blot

The cells were lysed with RIPA buffer (50 × 10^−3^ m pH 8.0 Tris‐HCl, 150 × 10^−3^ m NaCl, 5 × 10^−3^ m EDTA, 0.1% SDS, and 1% NP‐40) supplemented with a protease inhibitor cocktail. The protein concentration was measured by using a Bradford kit, and equal amounts of protein were loaded onto SDS‐PAGE gels. Primary antibodies were used against the following proteins: BCKDK (Santa Cruz, sc‐374424), RNF8 (Abcam, ab234977), p‐RNF8^S157^ (Abclonal, E19977), RAD51 (Proteintech, 67024‐1‐Ig), Ku70 (Proteintech, 10723‐1‐AP), p‐RPA32 (Bethyl, A300‐245A‐M), RPA32 (Proteintech, 10412‐1‐AP), γH2A.X (BBI, D55127‐0025; CST, 9718S), H2A.X (Proteintech, 10856‐1‐AP), RAD50 (Sangon Biotech, D160725‐0025), RAD52 (Sangon Biotech, D160724‐0025), p‐BCKDHA (Abcam, ab200577), Flag‐tag (Sigma, F1804), and β‐actin (Proteintech, 66009‐1‐Ig). Horseradish peroxidase‐conjugated anti‐rabbit (Bio‐Rad, 170–6515) and anti‐mouse (Bio‐Rad, 170–6516) secondary antibodies were applied. Signals were detected using Western ECL Substrate (Tanon, 180–501).

### qRT‐PCR

Total RNA was collected by using TRIzol reagent (Ambion) and reverse transcribed it with HiScript II 1st Strand cDNA Synthesis Kit (Vazyme, RC112). qRT‐PCR was performed using AceQ qPCR SYBR Green Master Mix (Vazyme, R323) on a Bio‐Rad iCycler. Primer sequences are provided in Table S5 (Supporting Information). mRNA levels in all samples were normalized to 18S rRNA.

### Immunoprecipitation assay

MDA‐468 or HEK293T cells were lysed in a buffer containing 20 × 10^−3^ m Tris‐HCl (pH 7.5), 150 × 10^−3^ m NaCl, 2 × 10^−3^ m EDTA, 1.5 × 10^−3^ m MgCl_2_, 1% NP‐40, a protease inhibitor cocktail and phosphatase inhibitors. The lysates were incubated on ice for 2 h and then centrifuged at 12 000 rpm for 10 min at 4 °C. The supernatants were pre‐cleared with protein A/G‐Sepharose beads for 2 h at 4 °C. After pre‐clearing, the supernatants were incubated with the indicated antibody for 8–12 h at 4 °C, followed by a 2 h incubation with protein A/G‐Sepharose beads at 4 °C. The beads were washed five times with IP buffer and the protein samples were resolved by SDS‐PAGE. For nuclear proteomics, Olaparib resistant MAD‐468, MDA‐231, and SUM149PT cells and extracted nuclear proteins were collected. Then, the peptides were analyzed on an QE‐plus mass spectrometer (Thermo Fisher Scientific, Waltham, MA, USA).

### Ubiquitination Assay

HEK293T cells were co‐transfected with hemagglutinin‐tagged ubiquitin (HA‐Ub) and RAD51‐3xFlag. After a 40‐h incubation, the proteasome inhibitor MG132 (10 × 10^−6^ m; Sigma, C2211) was added to the culture medium for 6–8 h. Then, cells were collected and proteins were extracted, and equal amounts of cell lysates were immunoprecipitated with an anti‐Flag‐M2 antibody (Sigma, F1804). Finally, the samples were subjected to SDS‐PAGE and immunoblotting with an anti‐HA antibody (Sigma, H3663).

### In Vitro Ubiquitination Assay

*Escherichia coli* BL21 cells (Beijing Tsingke Biotech Co., Ltd.) separately transformed with His‐tag H2A.X, His‐tag RAD51, Flag‐tag UBC13, GST‐tag RNF8^WT^, GST‐tag RNF8^S157A^, and GST‐tag RNF8^S157D^ were incubated and induced by IPTG as described above, and the cell extracts were used for protein purification with anti‐his magnetic beads (BeaverBeads; 70501‐100), anti‐flag magnetic beads (BeaverBeads; 22206‐1) and glutathione beads, respectively. The ubiquitination assay kit containing Ub thioester/conjugation initiation reagents was purchased from Abcam (ab139467). A 50 µL reaction mixture contained E1, Ub, Mg^2+^, ATP, reaction buffer from the kit, plus Flag‐tag UBC13 (10 µg), GST‐tag RNF8^WT^, GST‐tag RNF8^S157A^, GST‐tag RNF8^S157D^ (10 µg), and substrate (10 µg, His‐tag H2A.X or His‐tag RAD51) prepared from this study. Conjugation reactions were performed at 37 °C for 4 h according to manufacturer's instructions, followed by running of 12% SDS‐PAGE and Western blotting using anti‐Ub antibodies (Abclonal: A19686).

### Analysis of Efficiency of HRR and NHEJ

U2OS cells seeded at a density of 3 × 10^6^ cells. Then, U2OS cells transfected with pCBA‐SceI and pDR‐GFP (two plasmids were commercially purchased Hunan Fenghui Biotechnology Co., Ltd). On day 2 after transfection, U2OS cells passaged and continued to infect other genes virus before collection for analysis of HRR efficiency on FACSverse (BD Biosciences). DDR system was also used to test the efficiency of HRR and NHEJ. Cells were transfected with pLCN‐DDR plasmid, pCAGGS‐DRR‐mCherry‐Donor‐EF1a‐BFP plasmid, and pCBASceI plasmid for 48 h before fluorescence‐activated cell sorting analysis (besides pCBASceI plasmid, the other two plasmids were commercially purchased Shanghai Biofeng company Co., Ltd). The NHEJ and HRR efficiency were calculated as percentage of GFP/BFP and mCherry/BFP positive cells by flow cytometry.

### Immunofluorescence Staining

For IF staining, cells were fixed with 4% paraformaldehyde for 20 min and then washed them in PBS. The cells were permeabilized with 0.1% Triton X‐100 for 20 min, followed by blocking with 3% BSA for 2 h at room temperature. Primary antibodies, including anti‐BCKDK (Santa Cruz, sc‐374424, antibody dilution ratio 1:50), anti‐γH2A.X (1:100), were diluted in the blocking solution and incubated them with the cells in a humidified chamber overnight at 4 °C. Anti‐mouse or rabbit secondary antibodies conjugated to 594 (1:200) were used. The cells were stained with DAPI (Sigma‐Aldrich) for 5 min to detect nuclei. Representative microphotographs were acquired using a LSM 980 laser scanning confocal microscope (Zeiss) and analyzed them with ZEN microscope imaging software (Zeiss).

### Comet Assay

Collected cells by centrifugation at 200 g and adjusted the concentration to 6 × 10^10^ cells mL. The cells were prepared at a ratio of 1:3 (v/v, cell volume: low melting point agarose volume) and dropped them onto the glass slide, and covered the slide with a cover. After solidification, the cover glass was carefully removed and placed it in neutral lysis buffer (2 m NaCl, 30 × 10^−3^ m EDTA, 10 × 10^−3^ m Tris, 0.01% Triton X‐100, 0.1% DMSO) overnight at 4 °C. Then, the glass slide was placed in a horizontal electrophoresis tank, and then electrophoresed for 20 min under electric field (25 V, 150 mA). After electrophoresis, neutralized with Tris HCl (pH 7.5) for 15 min, then added ethidium bromide (EB) dropwise in the dark and cover with a cover glass. The representative microphotographs were acquired using a LSM 980 laser scanning confocal microscope (Zeiss) and analyzed them with Image J software.

### Metaphase Spread

MDA‐468 cells were incubated with colcemid (0.1 µg mL^−1^, TargetMol USA) at 37 °C for 4 h. After incubation, cells were collected and swollen in prewarmed 75 × 10^−3^ m KCl at 37 °C for 30 min. After centrifuging, cells were fixed with Carnoy's buffer (v/v, 3:1(methanol: acetic acid)) at room temperature for 10 min. The cells were centrifuged for 5 min at 200 g, and then the supernatant was aspirated. The cells were dropped onto slides and dried for at least 10 min. Slides were stained with Giemsa solution.

### In Vitro Kinase Assay

Protein expression was induced in BL21 bacteria (Tsingke), and treated with 0.1 × 10^−3^ m isopropyl β‐D‐thiogalactoside (IPTG) overnight at 16 °C. His‐tagged protein (His‐BCKDK) was purified using Nickel column and GST‐tagged protein (GST‐RNF8) was purified using GSH‐conjugated agarose beads (BeaverBeads, 70601–5). The purified proteins were concentrated. For pull‐down assay, the reaction was in PBST buffer (137 × 10^−3^ m NaCI, 2.7 × 10^−3^ m KCl, 10 × 10^−3^ m Na_2_HPO_4_, 2 × 10^−3^ m KH2PO4, 0.1% Tween‐20, and 2 × 10^−3^ m EDTA). Proteins incubated in 4 °C for 30 min, washed with PBST buffer, and then Western blot analysis using an anti‐His or anti‐GST antibody. For in vitro kinase assay, the reaction was in kinase buffer (50 × 10^−3^ m NaCl, 2 × 10^−3^ m EGTA, 25 × 10^−3^ m HEPES (pH 7.2), 5 × 10^−3^ m MgSO_4_, and 1 × 10^−3^ m DTT) containing 5 × 10^−6^ m ATP, 10 µg of His‐tagged BCKDK, and 10 µg of GST‐tagged RNF8, followed by incubation for 30 min at 37 °C. Moreover, the kinase inhibitor library (no. L1600) used was purchased from TargetMol.

### Nuclear Isolation

MDA‐468 cells were suspended in ice‐cold buffer A (150 × 10^−3^ m NaCl, 1 × 10^−3^ m KH_2_PO_4_, 5 × 10^−3^ m MgCl_2_, 0.2 × 10^−3^ m DTT, 1 × 10^−3^ m PMSF, 5 × 10^−3^ m NEM and 0.6% Triton X‐100) and vortex cracking for 10 min. The nuclear fraction was obtained by centrifugation 1500 g for 10 min, and washed in buffer A without 0.6% Triton X‐100. Nuclear were pelleted centrifugation 1500 g for 10 min and lysed in buffer B (10 × 10^−3^ m 2‐ME, 50 × 10^−3^ m pH7.5 Tris‐HCl, 500 × 10^−3^ m NaCl, 1 × 10^−3^ m EDTA, 10% glycerol, and 0.2% NP40 and protease inhibitors). Then, the protein concentration was measured by Bradford assay kit.

### Chromatin Extract Isolation

The cells (2 × 10^6^) were collected and were resuspended in 200 µL of solution A (10 × 10^−3^ m HEPES (pH 7.9), 10 × 10^−3^ m KCl, 1.5 × 10^−3^ m MgCl₂, 0.34 m sucrose, 10% glycerol, 1 × 10^−3^ m dithiothreitol, 10 × 10^−3^ m NaF, 1 × 10^−3^ m Na₂VO₃, and protease inhibitors). Then, Triton X‐100 was added to a final concentration of 0.1% and the cells were incubated on ice for 5 min. The cytoplasmic proteins were separated from the nuclei by low‐speed centrifugation at 1300 g for 5 min. The isolated nuclei were lysed in 200 µL of solution B (3 × 10^−3^ m EDTA, 0.2 × 10^−3^ m EGTA, 1 × 10^−3^ m dithiothreitol, and protease inhibitors). The insoluble chromatin was collected by high‐speed centrifugation at 10 000 g for 10 min. The chromatin pellet was resuspended in lysis buffer.

### ChIP Assay

A ChIP assay was performed using an EZ‐ChIP kit (17‐371, Millipore) following the manufacturer's instructions. Briefly, in the data, it was demonstrated that BCKDK was recruited to DNA damage site through ChIP assay. Collected USOS cells transfected with pDR‐GFP and pCBASceI for 48 h. DNA was then immunoprecipitated using either control IgG or anti‐Flag antibody, followed by qPCR analysis. The specific primers used in PCR for the sequences that were 0.2 and 0.6 kb distant from I‐SceI induced DSBs were 5′‐GATCAGGCAGAGCAGGAACC‐3′ (forward) and 5′‐GAACAGCTCCTCGCCCTTGC‐3′ (reverse), and 5′‐TCCATCTCCAGCCTCGGGGCT‐3′ (forward) and 5′‐AGGCTCTAGAGCCGCCGGTCA‐3′ (reverse), respectively.

### Development of Breast Cancer Organoids

Breast cancer organoids were generated following previously described protocols with slight modifications.^[^ ^51^ ^]^ Briefly, tissues (0.5–1 cm^3^) were minced and were digested with 2.5 mg mL^−1^ collagenase D (Roche) and 0.1 mg mL^−1^ DNase (Sigma) at 37 °C for 2–3 h. Then, 5000–10 000 cells were seeded in 50 µL of BME2 (Basement Membrane Extract Type 2, PathClear) per well in a 24‐well plate. After the BME solidified, the tumoroid‐specific culture medium was added. Information about the breast cancer patients is provided in Table S6 (Supporting Information).

### Clinical Breast Cancer Tissue Specimens

Snap‐frozen breast cancer tissues and corresponding noncancerous tissues were collected, which were at least 2 cm from the tumor edge, from 14 patients with breast cancer at the First Affiliated Hospital of the University of Science and Technology of China (Permit No. 2022KY‐241). Formalin‐fixed, paraffin‐embedded primary breast cancer tissue specimens were also selected from 103 patients at random from the hospital's archives. Clinical data and pathological characteristics of the patients were recorded, including age, gender, tumor size, and lymph node involvement. Detailed patient data are presented in Table S7 (Supporting Information). Tumor clinical staging was defined according to the 8^th^ edition of the American Joint Committee on Cancer/International Anti‐Cancer Alliance TNM Classification System.^[^ ^44^ ^]^ The written informed consent obtained from the patients and approval obtained from the Institutional Research Ethics Committee of the First Affiliated Hospital of the University of Science and Technology of China to use these clinical materials for research purposes. All patients participated voluntarily and received no compensation.

### Immunohistochemical

IHC was performed as previously described.^[^ ^52^ ^]^ Samples with xylene were dewaxed and rehydrated with graded ethanol. After antigen retrieval, endogenous peroxidase activity was blocked by incubating sections with 0.3% hydrogen peroxide for 10 min. Then, sections in normal goat serum were preincubated for 15 min to prevent nonspecific staining. Subsequently, samples were incubated with BCKDK, p‐RNF8^S157^, RNF8, p‐RPA32, or RAD51 antibodies overnight at room temperature. Four hours later, the secondary antibody was applied for 4 h and the sections were incubated with DAB Chromogen dilution solution. The AxioVision Rel.4.6 computerized image analysis system was used for quantitative analysis of IHC staining, aided by an automatic measurement program (Carl Zeiss). The IHC staining was quantitatively analyzed using TissueFAXS (TissueGnostic) and HistoQuest (v 4.0) (TissueGnostic). Six different staining fields of each section were analyzed to determine the mean optical sdensity (MOD), and a *t*‐test was used to compare average MOD differences between groups.

### Animal Studies

All animal studies received approval from the Animal Research Ethics Committee of the University of Science and Technology of China (Permit No. USTCACUC24120122074). Mice were purchased from Beijing Vital River Laboratory Animal Technology Co., Ltd. For the in vivo xenograft experiment, 5 × 10^6^ MDA‐468 cells were subcutaneous injection into 5‐week‐old female BALB/c nude mice, followed by the indicated treatments. For Olaparib (no. T3015, TargetMol, USA) treatment, when tumor volume reached 100 mm,^[^ ^3^ ^]^ Olaparib dissolved in DMSO and diluted as follows: 10% stock, 40% PEG300, 5% Tween‐80, 45% water (v/v), and was injected intraperitoneally every 3 days, with a final concentration of 25 mg kg^−1^. For GSK180736A (no. T3513, TargetMol, USA) treatment, GSK180736A dissolved in DMSO and diluted as follows: 5% stock, 30% PEG300, 5% Tween‐80, 60% water (v/v), and was injected intraperitoneally every 3 days, with a final concentration of 1 mg kg^−1^. Tumors with calipers were measured every 3 days and tumor volumes were using the formula: Tumor volume (mm^3^) = length (mm) × width^[^ ^2^ ^]^ (mm) × 0.52 (in MMTV‐PyMT mouse TNBC model) or tumor volume (mm^3^) = length (mm) × width (mm) × depth (mm) × 0.52 (in vivo xenograft experiment). In the MMTV‐PyMT mouse TNBC model, mice were sacrificed approximately 3 or 4 months later. According to the experimental methods of MMTV‐PyMT mice,^[^ ^48^, ^53^, ^54^, ^55^ ^]^ the tumor volume and weight of the MMTV‐PyMT mice were measured. This work was committed to following ethical guidelines for all the animal experiments.

### Statistical Analysis

The data was presented as the mean ± S.D. or mean ± S.E.M of at least three independent experiments, as indicated. Chi‐square test was used for IHC staining comparisons among different clinical stages of patient specimens. Log‐rank test was used for survival analyses. Student's *t*‐test was used for comparisons between two groups and ANOVA for multiple comparisons (GraphPad Software). A *p*‐value less than 0.05 was considered statistically significant, ^*^ *p* < 0.05; ^**^ *p* < 0.01; ^***^ *p* < 0.001; not significant is displayed as n.s.

## Conflict of Interest

The authors declare no conflict of interest.

## Author contributions

H.L., J.F., and T.P. contributed equally to this work. H.Z. and D.H. conceived and supervised the study. H.L., H.Z., D.H., and P.G. designed the experiments. H.L., J.F., and T.P. conducted experiments and collected data. J.F., X.Y., and Q.M. performed experiments of breast cancer organoid. T.P., Z.Z., and J.L. provided clinical specimens and analysis. P.Z., L.Y., and Z.J. analyzed proteomics data. H.Z., D.H., and H.L. wrote the manuscript. All the authors read and approved the manuscript.

## Supporting information



Supporting Information

Supplemental Table 1

Supplemental Table 2

Supplemental Table 3

Supplemental Table 4

Supplemental Table 5

Supplemental Table 6

Supplemental Table 7

## Data Availability

The data that support the findings of this study are available in the supplementary material of this article.
