# Supplementary material for: Nuclear‐Localized BCKDK Facilitates Homologous Recombination Repair to Support Breast Cancer Progression and Therapy Resistance
Source: Adv Sci (Weinh). 2025 Apr 29;12(22):2416590. doi: 10.1002/advs.202416590 (PMC12165075; doi:10.1002/advs.202416590)
Supplement: Supplementary file 1 — Supporting Information [file ADVS-12-2416590-s005.pptx]

## Slide 1
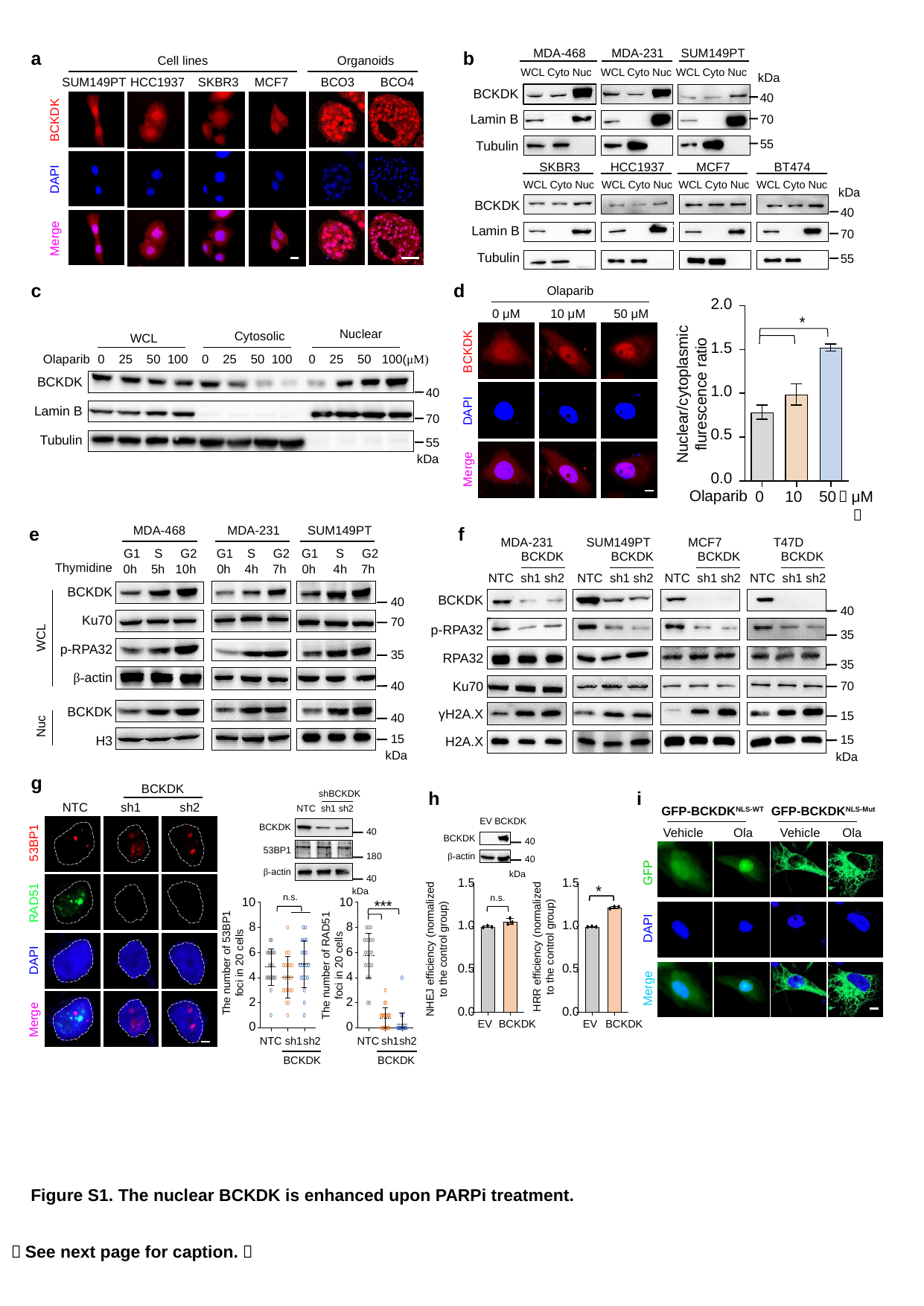

MDA-468
MDA-231
SUM149PT
WCL Cyto Nuc
WCL Cyto Nuc
WCL Cyto Nuc
kDa
BCKDK
40
Lamin B
70
55
Tubulin
a
b
Cell lines
SUM149PT
HCC1937
SKBR3
MCF7
BCKDK
DAPI
Merge
Organoids
BCO4
BCO3
SKBR3
HCC1937
MCF7
BT474
WCL Cyto Nuc
WCL Cyto Nuc
WCL Cyto Nuc
WCL Cyto Nuc
kDa
BCKDK
40
Lamin B
70
Tubulin
55
c
d
2.0
*
1.5
Nuclear/cytoplasmic flurescence ratio
1.0
0.5
0.0
Olaparib
0
10
50
（μM）
Olaparib
0 μM
10 μM
50 μM
BCKDK
DAPI
Merge
Nuclear
Cytosolic
WCL
Olaparib
0 25 50 100
0 25 50 100
0 25 50 100(μM)
BCKDK
Lamin B
Tubulin
kDa
40
70
55
MDA-468
MDA-231
SUM149PT
G1 S G2
G1 S G2
G1 S G2
Thymidine
0h 5h 10h
0h 4h 7h
0h 4h 7h
BCKDK
40
Ku70
70
WCL
p-RPA32
35
β-actin
40
BCKDK
40
Nuc
15
H3
kDa
e
f
MDA-231
SUM149PT
MCF7
T47D
BCKDK
BCKDK
BCKDK
BCKDK
 NTC sh1 sh2
 NTC sh1 sh2
 NTC sh1 sh2
 NTC sh1 sh2
BCKDK
40
p-RPA32
35
RPA32
35
70
Ku70
γH2A.X
15
15
H2A.X
kDa
g
BCKDK
NTC
sh1
53BP1
RAD51
DAPI
Merge
sh2
shBCKDK
 NTC sh1 sh2
BCKDK
40
53BP1
180
β-actin
40
kDa
h
i
GFP-BCKDKNLS-WT
GFP-BCKDKNLS-Mut
Vehicle
Ola
Vehicle
Ola
GFP
DAPI
Merge
 EV BCKDK
BCKDK
40
β-actin
40
kDa
1.5
1.0
0.5
0.0
EV
BCKDK
NHEJ efficiency (normalized to the control group)
n.s.
1.5
1.0
0.5
0.0
EV
BCKDK
HRR efficiency (normalized to the control group)
*
10
8
6
The number of 53BP1 foci in 20 cells
4
2
0
NTC
sh1
sh2
BCKDK
n.s.
10
8
6
The number of RAD51 foci in 20 cells
4
2
0
NTC
sh1
sh2
BCKDK
***
Figure S1. The nuclear BCKDK is enhanced upon PARPi treatment.
（See next page for caption.）

## Slide 2
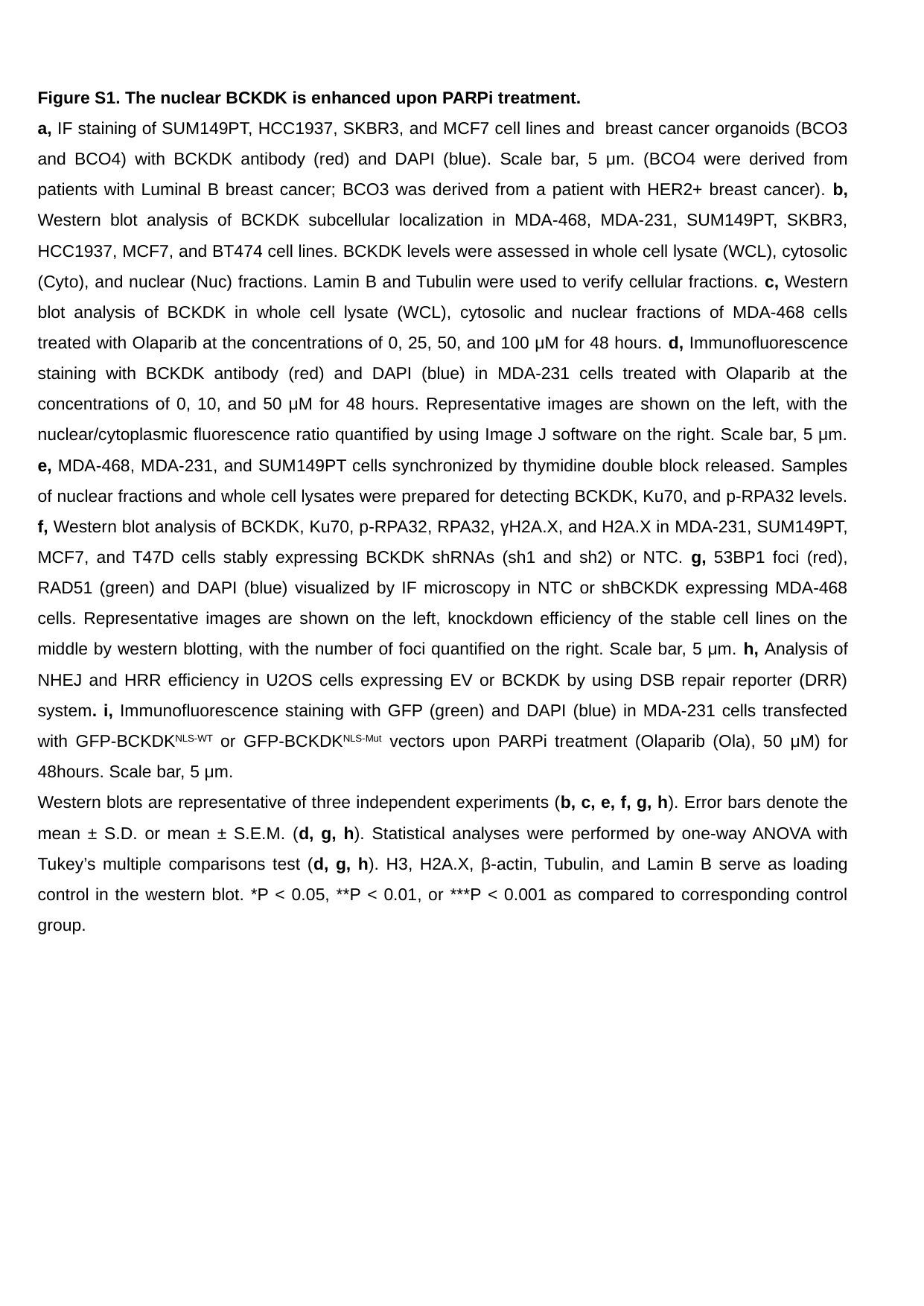

Figure S1. The nuclear BCKDK is enhanced upon PARPi treatment.
a, IF staining of SUM149PT, HCC1937, SKBR3, and MCF7 cell lines and breast cancer organoids (BCO3 and BCO4) with BCKDK antibody (red) and DAPI (blue). Scale bar, 5 μm. (BCO4 were derived from patients with Luminal B breast cancer; BCO3 was derived from a patient with HER2+ breast cancer). b, Western blot analysis of BCKDK subcellular localization in MDA-468, MDA-231, SUM149PT, SKBR3, HCC1937, MCF7, and BT474 cell lines. BCKDK levels were assessed in whole cell lysate (WCL), cytosolic (Cyto), and nuclear (Nuc) fractions. Lamin B and Tubulin were used to verify cellular fractions. c, Western blot analysis of BCKDK in whole cell lysate (WCL), cytosolic and nuclear fractions of MDA-468 cells treated with Olaparib at the concentrations of 0, 25, 50, and 100 μM for 48 hours. d, Immunofluorescence staining with BCKDK antibody (red) and DAPI (blue) in MDA-231 cells treated with Olaparib at the concentrations of 0, 10, and 50 μM for 48 hours. Representative images are shown on the left, with the nuclear/cytoplasmic fluorescence ratio quantified by using Image J software on the right. Scale bar, 5 μm. e, MDA-468, MDA-231, and SUM149PT cells synchronized by thymidine double block released. Samples of nuclear fractions and whole cell lysates were prepared for detecting BCKDK, Ku70, and p-RPA32 levels. f, Western blot analysis of BCKDK, Ku70, p-RPA32, RPA32, γH2A.X, and H2A.X in MDA-231, SUM149PT, MCF7, and T47D cells stably expressing BCKDK shRNAs (sh1 and sh2) or NTC. g, 53BP1 foci (red), RAD51 (green) and DAPI (blue) visualized by IF microscopy in NTC or shBCKDK expressing MDA-468 cells. Representative images are shown on the left, knockdown efficiency of the stable cell lines on the middle by western blotting, with the number of foci quantified on the right. Scale bar, 5 μm. h, Analysis of NHEJ and HRR efficiency in U2OS cells expressing EV or BCKDK by using DSB repair reporter (DRR) system. i, Immunofluorescence staining with GFP (green) and DAPI (blue) in MDA-231 cells transfected with GFP-BCKDKNLS-WT or GFP-BCKDKNLS-Mut vectors upon PARPi treatment (Olaparib (Ola), 50 μM) for 48hours. Scale bar, 5 μm.
Western blots are representative of three independent experiments (b, c, e, f, g, h). Error bars denote the mean ± S.D. or mean ± S.E.M. (d, g, h). Statistical analyses were performed by one-way ANOVA with Tukey’s multiple comparisons test (d, g, h). H3, H2A.X, β-actin, Tubulin, and Lamin B serve as loading control in the western blot. *P < 0.05, **P < 0.01, or ***P < 0.001 as compared to corresponding control group.

## Slide 3
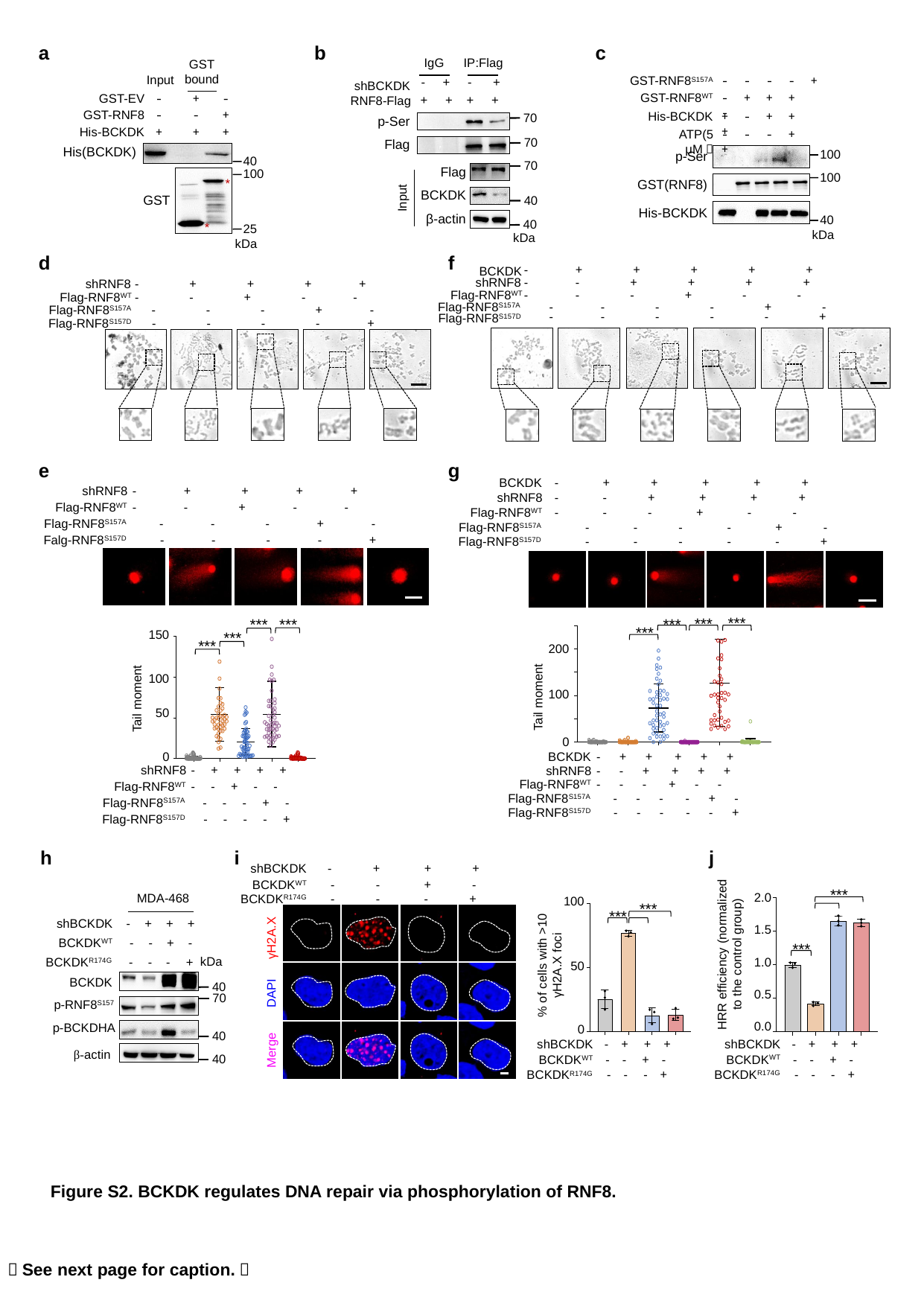

a
b
c
IgG
IP:Flag
 - + - +
shBCKDK
 + + + +
RNF8-Flag
70
p-Ser
70
70
Flag
BCKDK
Input
40
β-actin
40
kDa
Flag
GST
bound
Input
GST-EV
- + -
GST-RNF8
- - +
His-BCKDK
+ + +
His(BCKDK)
40
100
*
GST
*
25
kDa
GST-RNF8S157A
- - - - +
GST-RNF8WT
- + + + -
+ - + + +
- - - + +
ATP(5 μM）
100
p-Ser
100
GST(RNF8)
His-BCKDK
40
His-BCKDK
kDa
d
f
- + + + + +
BCKDK
shRNF8
- - + + + +
Flag-RNF8WT
- - - + - -
Flag-RNF8S157A
- - - - + -
- - - - - +
Flag-RNF8S157D
shRNF8
- + + + +
Flag-RNF8WT
- - + - -
Flag-RNF8S157A
- - - + -
Flag-RNF8S157D
- - - - +
e
g
BCKDK
- + + + + +
shRNF8
- + + + +
Flag-RNF8WT
- - + - -
Flag-RNF8S157A
- - - + -
Falg-RNF8S157D
- - - - +
shRNF8
- - + + + +
Flag-RNF8WT
- - - + - -
Flag-RNF8S157A
- - - - + -
Flag-RNF8S157D
- - - - - +
200
100
Tail moment
0
BCKDK
- + + + + +
shRNF8
- - + + + +
Flag-RNF8WT
- - - + - -
Flag-RNF8S157A
- - - - + -
***
***
***
***
***
***
150
***
***
100
Tail moment
50
0
shRNF8
- + + + +
Flag-RNF8WT
- - + - -
Flag-RNF8S157A
- - - + -
Flag-RNF8S157D
- - - - +
Flag-RNF8S157D
- - - - - +
h
i
j
shBCKDK
- + + +
BCKDKWT
- - + -
BCKDKR174G
- - - +
γH2A.X
DAPI
Merge
***
2.0
MDA-468
100
***
***
shBCKDK
- + + +
1.5
BCKDKWT
- - + -
***
HRR efficiency (normalized to the control group)
% of cells with >10 γH2A.X foci
kDa
1.0
BCKDKR174G
- - - +
50
BCKDK
40
0.5
70
p-RNF8S157
0.0
shBCKDK
- + + +
BCKDKWT
- - + -
BCKDKR174G
- - - +
p-BCKDHA
0
40
shBCKDK
- + + +
BCKDKWT
- - + -
BCKDKR174G
- - - +
β-actin
40
Figure S2. BCKDK regulates DNA repair via phosphorylation of RNF8.
（See next page for caption.）

## Slide 4
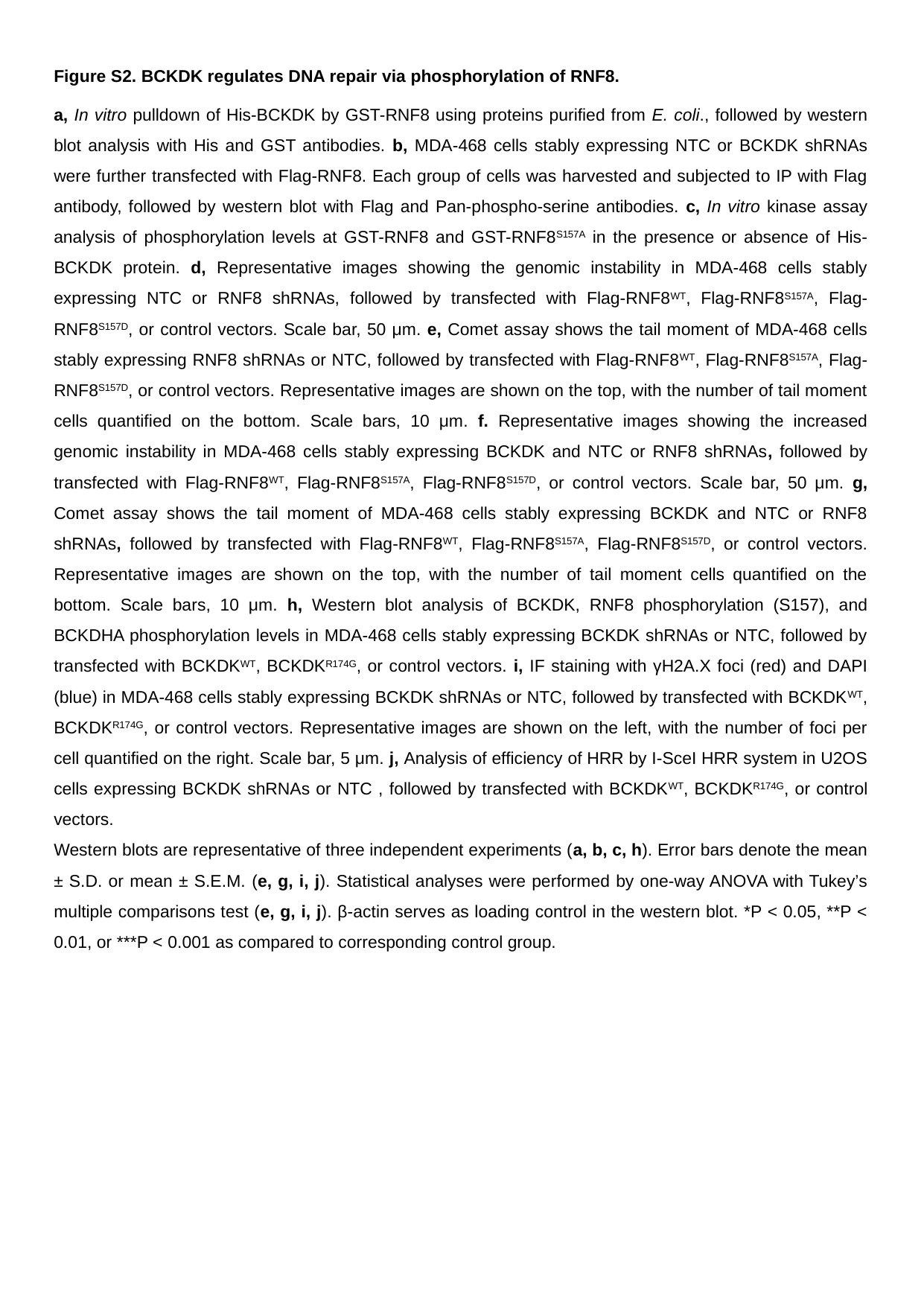

Figure S2. BCKDK regulates DNA repair via phosphorylation of RNF8.
a, In vitro pulldown of His-BCKDK by GST-RNF8 using proteins purified from E. coli., followed by western blot analysis with His and GST antibodies. b, MDA-468 cells stably expressing NTC or BCKDK shRNAs were further transfected with Flag-RNF8. Each group of cells was harvested and subjected to IP with Flag antibody, followed by western blot with Flag and Pan-phospho-serine antibodies. c, In vitro kinase assay analysis of phosphorylation levels at GST-RNF8 and GST-RNF8S157A in the presence or absence of His-BCKDK protein. d, Representative images showing the genomic instability in MDA-468 cells stably expressing NTC or RNF8 shRNAs, followed by transfected with Flag-RNF8WT, Flag-RNF8S157A, Flag-RNF8S157D, or control vectors. Scale bar, 50 μm. e, Comet assay shows the tail moment of MDA-468 cells stably expressing RNF8 shRNAs or NTC, followed by transfected with Flag-RNF8WT, Flag-RNF8S157A, Flag-RNF8S157D, or control vectors. Representative images are shown on the top, with the number of tail moment cells quantified on the bottom. Scale bars, 10 μm. f. Representative images showing the increased genomic instability in MDA-468 cells stably expressing BCKDK and NTC or RNF8 shRNAs, followed by transfected with Flag-RNF8WT, Flag-RNF8S157A, Flag-RNF8S157D, or control vectors. Scale bar, 50 μm. g, Comet assay shows the tail moment of MDA-468 cells stably expressing BCKDK and NTC or RNF8 shRNAs, followed by transfected with Flag-RNF8WT, Flag-RNF8S157A, Flag-RNF8S157D, or control vectors. Representative images are shown on the top, with the number of tail moment cells quantified on the bottom. Scale bars, 10 μm. h, Western blot analysis of BCKDK, RNF8 phosphorylation (S157), and BCKDHA phosphorylation levels in MDA-468 cells stably expressing BCKDK shRNAs or NTC, followed by transfected with BCKDKWT, BCKDKR174G, or control vectors. i, IF staining with γH2A.X foci (red) and DAPI (blue) in MDA-468 cells stably expressing BCKDK shRNAs or NTC, followed by transfected with BCKDKWT, BCKDKR174G, or control vectors. Representative images are shown on the left, with the number of foci per cell quantified on the right. Scale bar, 5 μm. j, Analysis of efficiency of HRR by I-SceI HRR system in U2OS cells expressing BCKDK shRNAs or NTC , followed by transfected with BCKDKWT, BCKDKR174G, or control vectors.
Western blots are representative of three independent experiments (a, b, c, h). Error bars denote the mean ± S.D. or mean ± S.E.M. (e, g, i, j). Statistical analyses were performed by one-way ANOVA with Tukey’s multiple comparisons test (e, g, i, j). β-actin serves as loading control in the western blot. *P < 0.05, **P < 0.01, or ***P < 0.001 as compared to corresponding control group.

## Slide 5
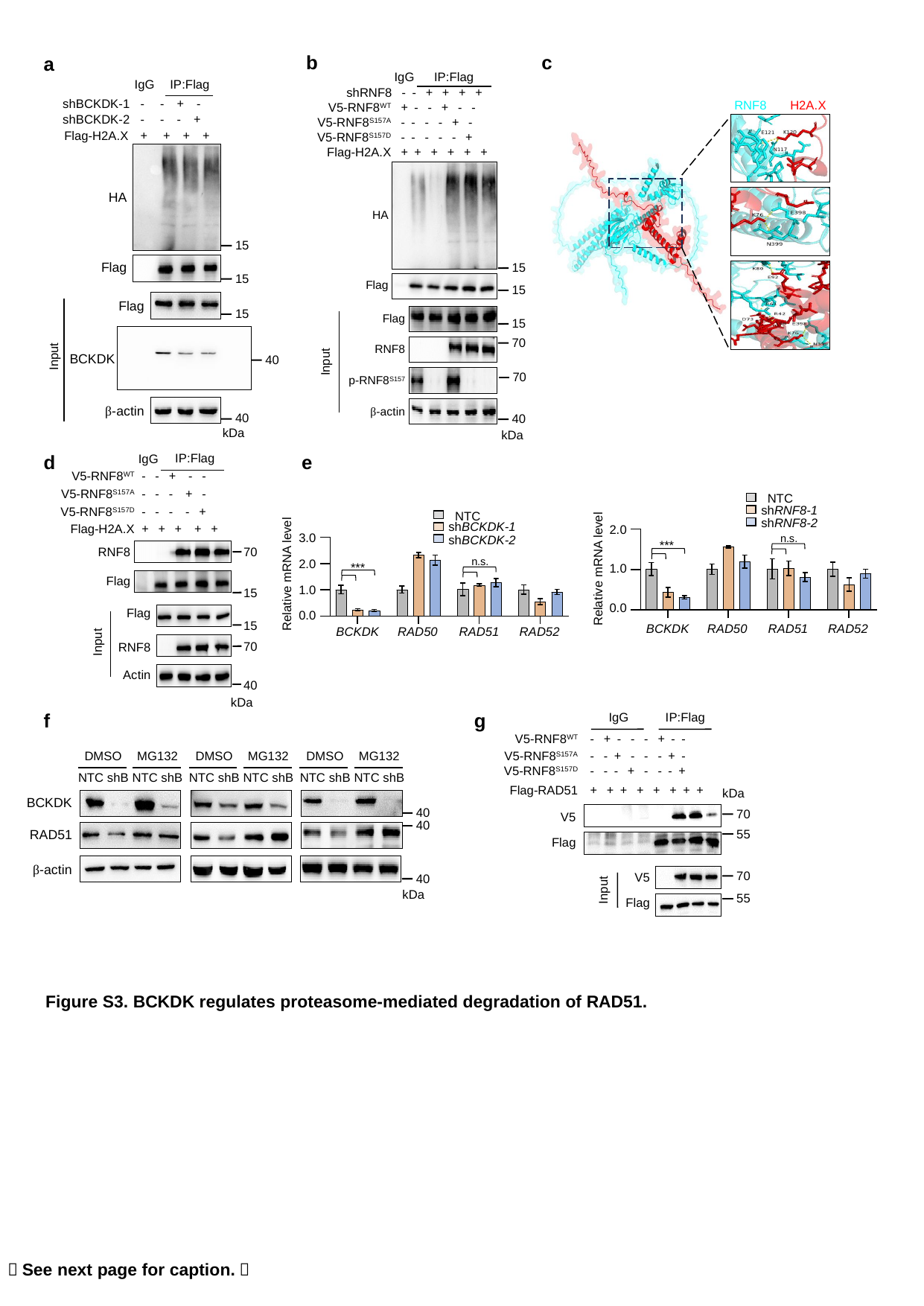

b
c
a
IgG
IP:Flag
shRNF8
- - + + + +
V5-RNF8WT
+ - - + - -
V5-RNF8S157A
- - - - + -
V5-RNF8S157D
- - - - - +
Flag-H2A.X
+ + + + + +
HA
15
Flag
15
Flag
15
70
RNF8
Input
70
p-RNF8S157
β-actin
40
kDa
IgG
IP:Flag
shBCKDK-1
- - + -
shBCKDK-2
- - - +
Flag-H2A.X
+ + + +
HA
15
Flag
15
Flag
15
BCKDK
40
Input
β-actin
40
kDa
RNF8
H2A.X
IP:Flag
V5-RNF8WT
- - + - -
V5-RNF8S157A
- - - + -
V5-RNF8S157D
- - - - +
Flag-H2A.X
+ + + + +
RNF8
70
Flag
15
Flag
15
Input
70
RNF8
Actin
40
kDa
IgG
d
e
NTC
shRNF8-1
shRNF8-2
2.0
n.s.
***
Relative mRNA level
1.0
0.0
BCKDK
RAD50
RAD51
RAD52
NTC
shBCKDK-1
shBCKDK-2
3.0
2.0
Relative mRNA level
1.0
0.0
BCKDK
RAD50
RAD51
RAD52
n.s.
***
f
IgG
IP:Flag
V5-RNF8WT
- + - - - + - -
V5-RNF8S157A
- - + - - - + -
V5-RNF8S157D
- - - + - - - +
Flag-RAD51
+ + + + + + + +
kDa
70
V5
55
Flag
70
V5
Input
55
Flag
g
DMSO
MG132
DMSO
MG132
DMSO
MG132
NTC shB NTC shB
NTC shB NTC shB
NTC shB NTC shB
BCKDK
40
40
RAD51
β-actin
40
kDa
Figure S3. BCKDK regulates proteasome-mediated degradation of RAD51.
（See next page for caption.）

## Slide 6
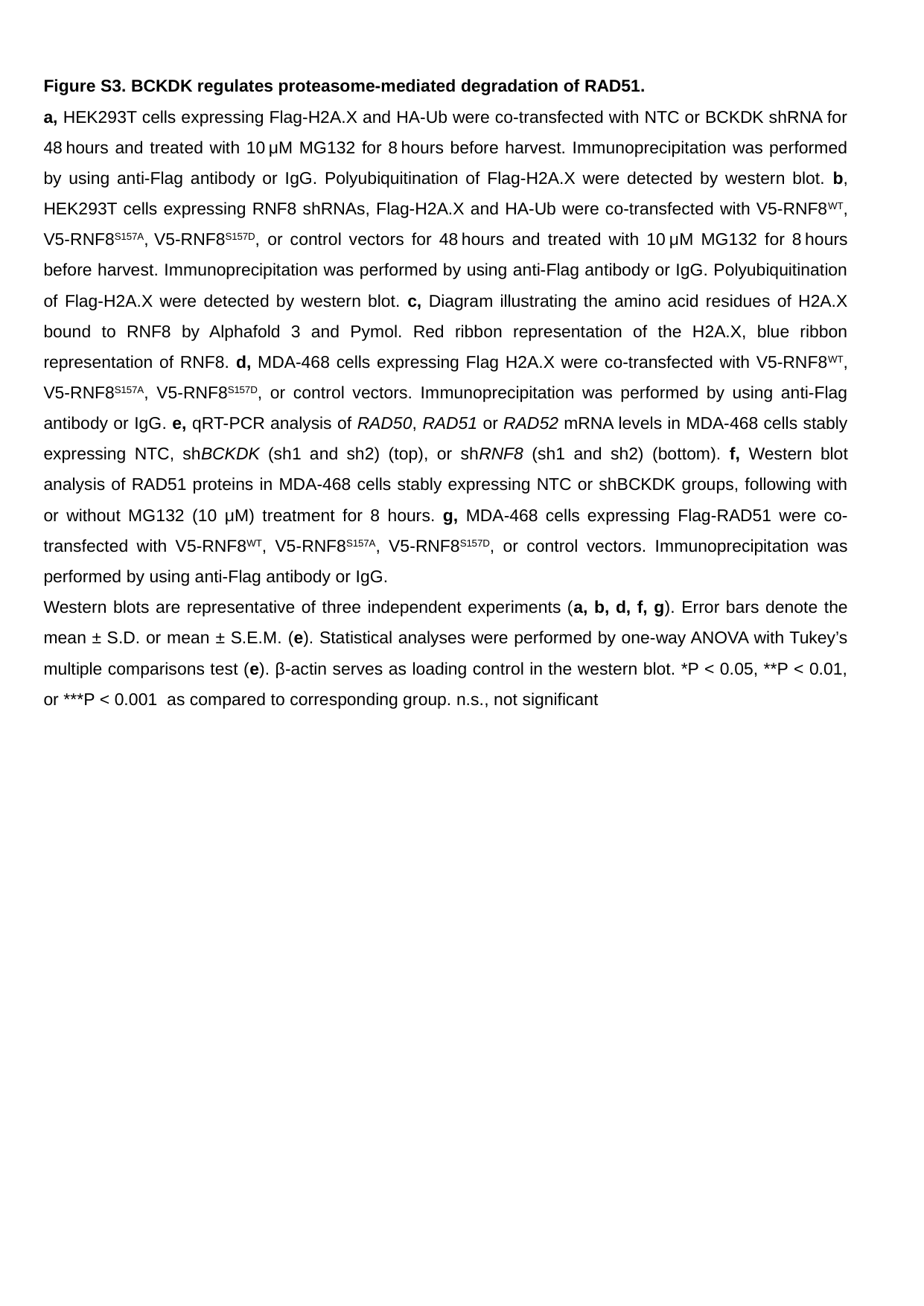

Figure S3. BCKDK regulates proteasome-mediated degradation of RAD51.
a, HEK293T cells expressing Flag-H2A.X and HA-Ub were co-transfected with NTC or BCKDK shRNA for 48 hours and treated with 10 μM MG132 for 8 hours before harvest. Immunoprecipitation was performed by using anti-Flag antibody or IgG. Polyubiquitination of Flag-H2A.X were detected by western blot. b, HEK293T cells expressing RNF8 shRNAs, Flag-H2A.X and HA-Ub were co-transfected with V5-RNF8WT, V5-RNF8S157A, V5-RNF8S157D, or control vectors for 48 hours and treated with 10 μM MG132 for 8 hours before harvest. Immunoprecipitation was performed by using anti-Flag antibody or IgG. Polyubiquitination of Flag-H2A.X were detected by western blot. c, Diagram illustrating the amino acid residues of H2A.X bound to RNF8 by Alphafold 3 and Pymol. Red ribbon representation of the H2A.X, blue ribbon representation of RNF8. d, MDA-468 cells expressing Flag H2A.X were co-transfected with V5-RNF8WT, V5-RNF8S157A, V5-RNF8S157D, or control vectors. Immunoprecipitation was performed by using anti-Flag antibody or IgG. e, qRT-PCR analysis of RAD50, RAD51 or RAD52 mRNA levels in MDA-468 cells stably expressing NTC, shBCKDK (sh1 and sh2) (top), or shRNF8 (sh1 and sh2) (bottom). f, Western blot analysis of RAD51 proteins in MDA-468 cells stably expressing NTC or shBCKDK groups, following with or without MG132 (10 μM) treatment for 8 hours. g, MDA-468 cells expressing Flag-RAD51 were co-transfected with V5-RNF8WT, V5-RNF8S157A, V5-RNF8S157D, or control vectors. Immunoprecipitation was performed by using anti-Flag antibody or IgG.
Western blots are representative of three independent experiments (a, b, d, f, g). Error bars denote the mean ± S.D. or mean ± S.E.M. (e). Statistical analyses were performed by one-way ANOVA with Tukey’s multiple comparisons test (e). β-actin serves as loading control in the western blot. *P < 0.05, **P < 0.01, or ***P < 0.001 as compared to corresponding group. n.s., not significant

## Slide 7
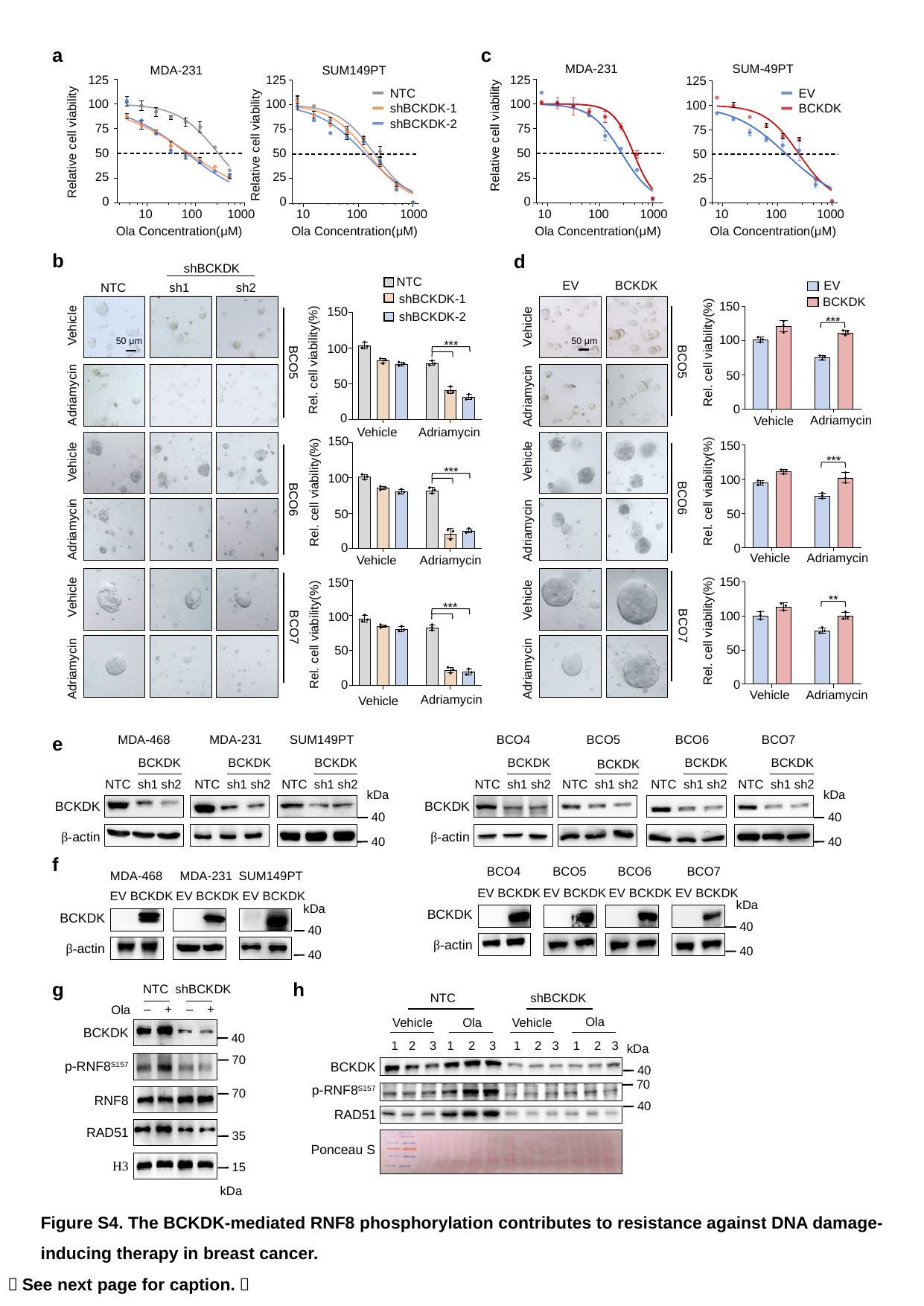

c
a
Relative cell viability
125
100
75
50
25
0
10
100
1000
Ola Concentration(μM)
Relative cell viability
125
100
75
50
25
0
10
100
1000
Ola Concentration(μM)
MDA-231
SUM-49PT
MDA-231
SUM149PT
Relative cell viability
125
125
NTC
shBCKDK-1
shBCKDK-2
EV
BCKDK
100
100
75
75
50
50
25
25
0
0
10
100
1000
10
100
1000
Ola Concentration(μM)
Ola Concentration(μM)
b
d
shBCKDK
NTC
shBCKDK-1
shBCKDK-2
EV
BCKDK
EV
BCKDK
NTC
sh1
sh2
150
***
100
Rel. cell viability(%)
50
0
Vehicle
Adriamycin
150
Vehicle
Vehicle
50 μm
50 μm
***
100
Rel. cell viability(%)
BCO5
BCO5
50
Adriamycin
Adriamycin
0
150
***
100
50
0
Vehicle
Adriamycin
Rel. cell viability(%)
Vehicle
Adriamycin
150
Vehicle
Vehicle
***
100
Rel. cell viability(%)
BCO6
BCO6
50
Adriamycin
Adriamycin
0
Vehicle
Adriamycin
**
50
0
Vehicle
Adriamycin
150
150
Vehicle
Vehicle
***
100
100
BCO7
BCO7
Rel. cell viability(%)
Rel. cell viability(%)
50
Adriamycin
Adriamycin
0
Adriamycin
Vehicle
MDA-468
MDA-231
SUM149PT
BCO4
BCO5
BCO6
BCO7
BCKDK
BCKDK
BCKDK
BCKDK
BCKDK
BCKDK
BCKDK
 NTC sh1 sh2
 NTC sh1 sh2
 NTC sh1 sh2
 NTC sh1 sh2
 NTC sh1 sh2
 NTC sh1 sh2
 NTC sh1 sh2
kDa
kDa
BCKDK
BCKDK
40
40
β-actin
β-actin
40
40
e
f
BCO4
BCO5
BCO6
BCO7
 EV BCKDK
 EV BCKDK
 EV BCKDK
 EV BCKDK
kDa
BCKDK
β-actin
40
40
MDA-468
MDA-231
SUM149PT
 EV BCKDK
 EV BCKDK
 EV BCKDK
kDa
BCKDK
40
β-actin
40
g
h
NTC
shBCKDK
NTC
shBCKDK
Ola
Vehicle
Ola
Vehicle
1 2 3 1 2 3 1 2 3 1 2 3
kDa
BCKDK
40
70
p-RNF8S157
40
RAD51
Ponceau S
 – + – +
Ola
BCKDK
40
70
p-RNF8S157
70
RNF8
RAD51
35
H3
15
kDa
Figure S4. The BCKDK-mediated RNF8 phosphorylation contributes to resistance against DNA damage-inducing therapy in breast cancer.
（See next page for caption.）

## Slide 8
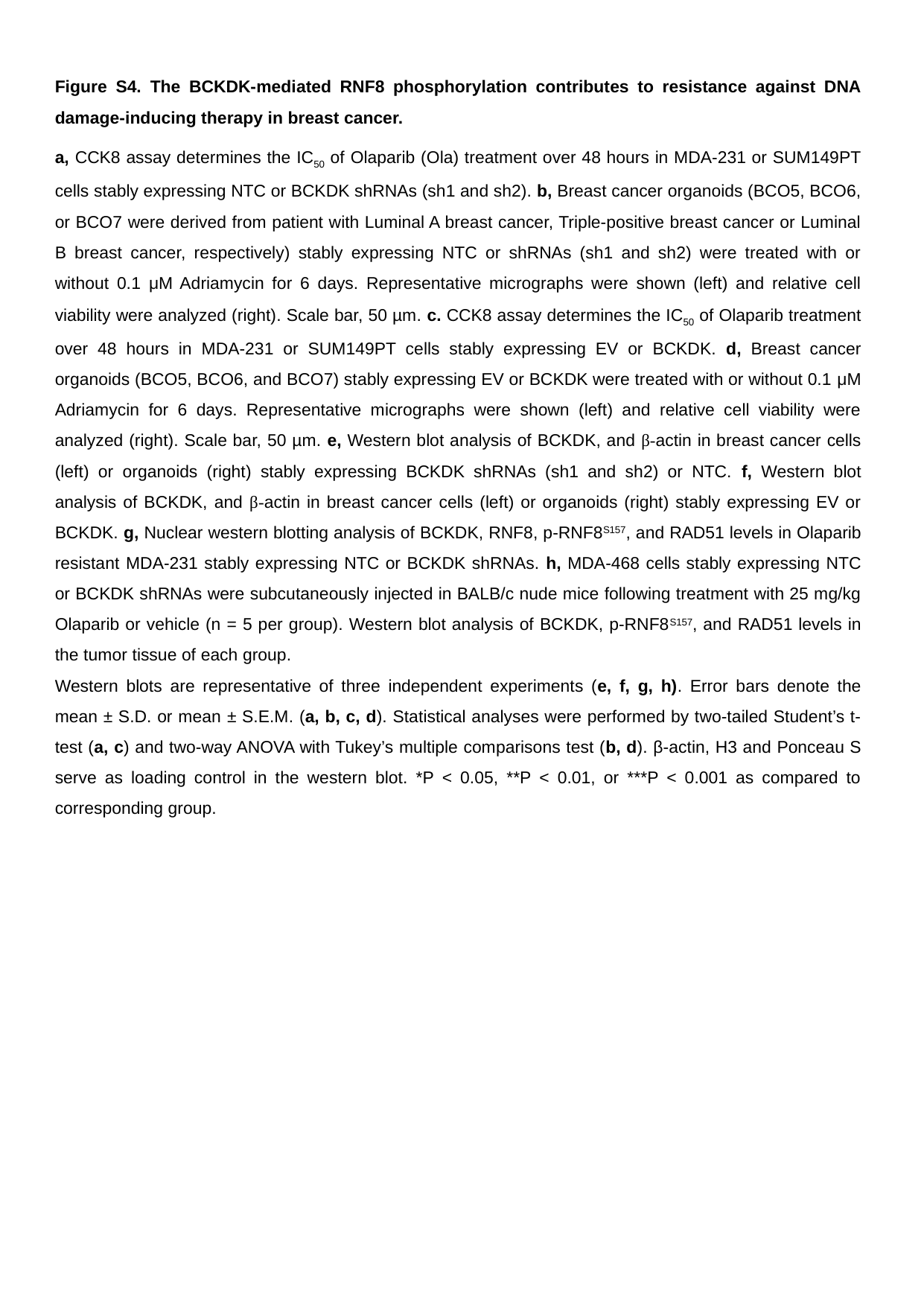

Figure S4. The BCKDK-mediated RNF8 phosphorylation contributes to resistance against DNA damage-inducing therapy in breast cancer.
a, CCK8 assay determines the IC50 of Olaparib (Ola) treatment over 48 hours in MDA-231 or SUM149PT cells stably expressing NTC or BCKDK shRNAs (sh1 and sh2). b, Breast cancer organoids (BCO5, BCO6, or BCO7 were derived from patient with Luminal A breast cancer, Triple-positive breast cancer or Luminal B breast cancer, respectively) stably expressing NTC or shRNAs (sh1 and sh2) were treated with or without 0.1 μM Adriamycin for 6 days. Representative micrographs were shown (left) and relative cell viability were analyzed (right). Scale bar, 50 µm. c. CCK8 assay determines the IC50 of Olaparib treatment over 48 hours in MDA-231 or SUM149PT cells stably expressing EV or BCKDK. d, Breast cancer organoids (BCO5, BCO6, and BCO7) stably expressing EV or BCKDK were treated with or without 0.1 μM Adriamycin for 6 days. Representative micrographs were shown (left) and relative cell viability were analyzed (right). Scale bar, 50 µm. e, Western blot analysis of BCKDK, and β-actin in breast cancer cells (left) or organoids (right) stably expressing BCKDK shRNAs (sh1 and sh2) or NTC. f, Western blot analysis of BCKDK, and β-actin in breast cancer cells (left) or organoids (right) stably expressing EV or BCKDK. g, Nuclear western blotting analysis of BCKDK, RNF8, p-RNF8S157, and RAD51 levels in Olaparib resistant MDA-231 stably expressing NTC or BCKDK shRNAs. h, MDA-468 cells stably expressing NTC or BCKDK shRNAs were subcutaneously injected in BALB/c nude mice following treatment with 25 mg/kg Olaparib or vehicle (n = 5 per group). Western blot analysis of BCKDK, p-RNF8S157, and RAD51 levels in the tumor tissue of each group.
Western blots are representative of three independent experiments (e, f, g, h). Error bars denote the mean ± S.D. or mean ± S.E.M. (a, b, c, d). Statistical analyses were performed by two-tailed Student’s t-test (a, c) and two-way ANOVA with Tukey’s multiple comparisons test (b, d). β-actin, H3 and Ponceau S serve as loading control in the western blot. *P < 0.05, **P < 0.01, or ***P < 0.001 as compared to corresponding group.

## Slide 9
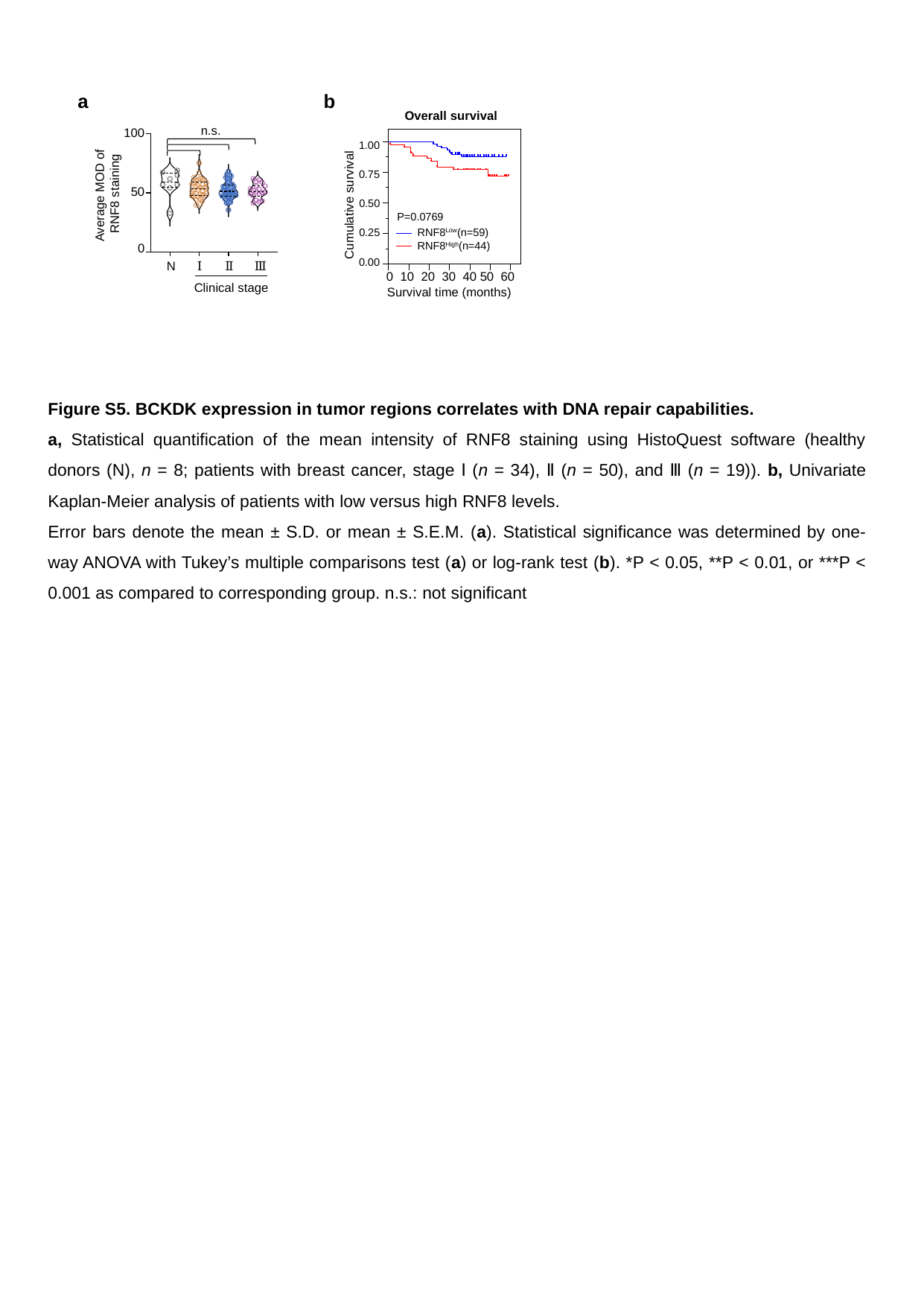

Overall survival
1.00
0.75
0.50
Cumulative survival
0.25
0.00
0 10 20 30 40 50 60
Survival time (months)
P=0.0769
RNF8Low(n=59)
RNF8High(n=44)
a
b
Average MOD of
 RNF8 staining
N
Ⅰ
Ⅱ
Ⅲ
Clinical stage
100
50
0
n.s.
Figure S5. BCKDK expression in tumor regions correlates with DNA repair capabilities.
a, Statistical quantification of the mean intensity of RNF8 staining using HistoQuest software (healthy donors (N), n = 8; patients with breast cancer, stage Ⅰ (n = 34), Ⅱ (n = 50), and Ⅲ (n = 19)). b, Univariate Kaplan-Meier analysis of patients with low versus high RNF8 levels.
Error bars denote the mean ± S.D. or mean ± S.E.M. (a). Statistical significance was determined by one-way ANOVA with Tukey’s multiple comparisons test (a) or log-rank test (b). *P < 0.05, **P < 0.01, or ***P < 0.001 as compared to corresponding group. n.s.: not significant

## Slide 10
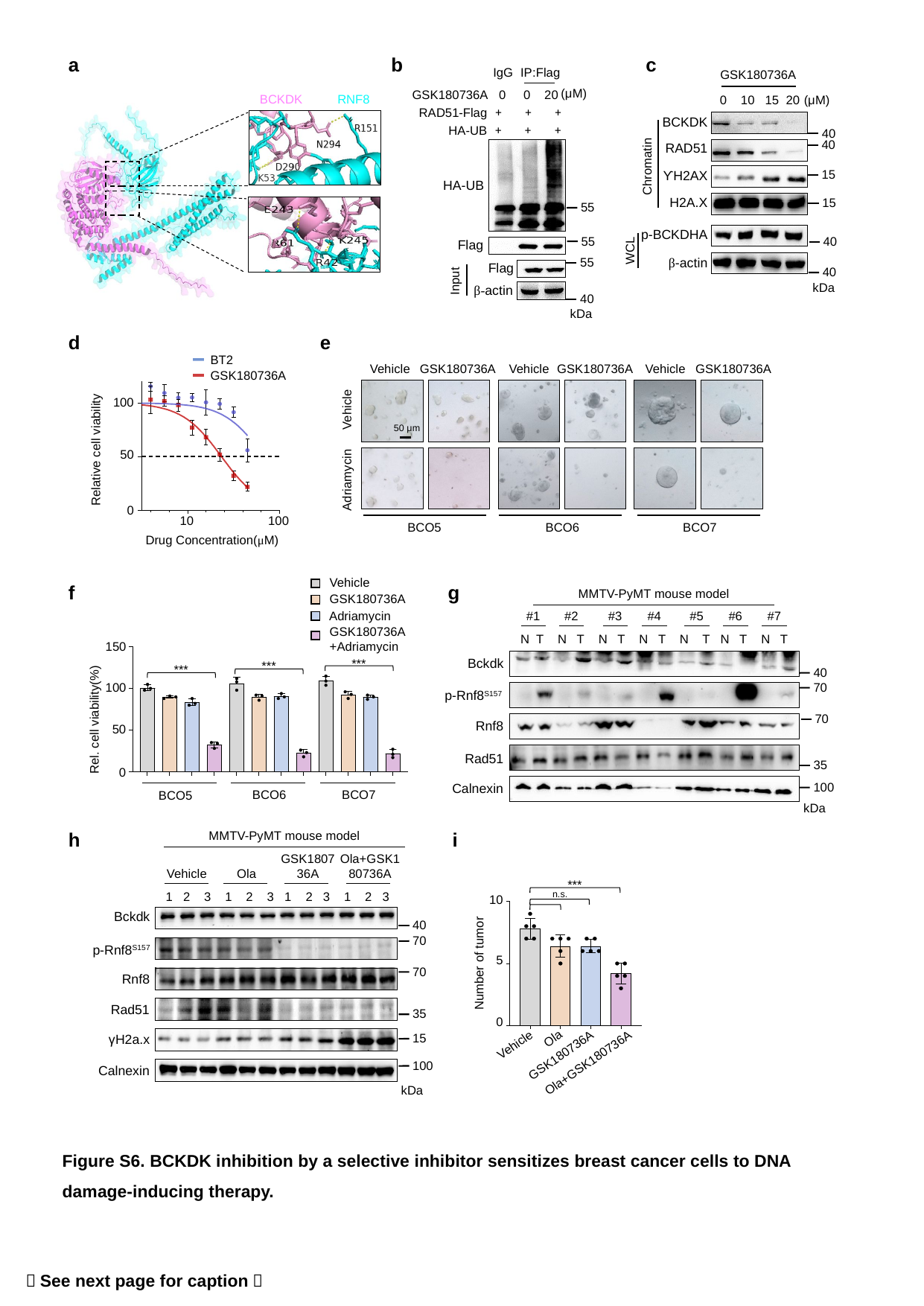

a
b
c
IgG
IP:Flag
(μM)
GSK180736A
 0 0 20
RAD51-Flag
+ + +
HA-UB
+ + +
HA-UB
55
55
Flag
55
Flag
Input
β-actin
40
kDa
GSK180736A
(μM)
 0 10 15 20
BCKDK
RAD51
Chromatin
ϒH2AX
H2A.X
p-BCKDHA
WCL
β-actin
40
40
15
15
40
40
kDa
BCKDK
RNF8
d
e
Relative cell viability
Drug Concentration(μM)
BT2
GSK180736A
100
50
0
10
100
Vehicle
GSK180736A
Vehicle
GSK180736A
Vehicle
GSK180736A
Vehicle
50 μm
Adriamycin
BCO6
BCO7
BCO5
Vehicle
GSK180736A
Adriamycin
GSK180736A
+Adriamycin
f
g
MMTV-PyMT mouse model
#1
#2
#3
#4
#5
#6
#7
N T N T N T N T N T N T N T
Bckdk
40
70
p-Rnf8S157
70
Rnf8
Rad51
35
100
Calnexin
kDa
Rel. cell viability(%)
BCO6
BCO7
BCO5
150
***
***
***
100
50
0
MMTV-PyMT mouse model
GSK180736A
Ola+GSK180736A
Vehicle
Ola
1 2 3 1 2 3 1 2 3 1 2 3
Bckdk
40
70
p-Rnf8S157
70
Rnf8
Rad51
35
15
γH2a.x
100
Calnexin
kDa
h
i
***
n.s.
10
5
Number of tumor
0
Ola
Vehicle
GSK180736A
Ola+GSK180736A
Figure S6. BCKDK inhibition by a selective inhibitor sensitizes breast cancer cells to DNA damage-inducing therapy.
（See next page for caption）

## Slide 11
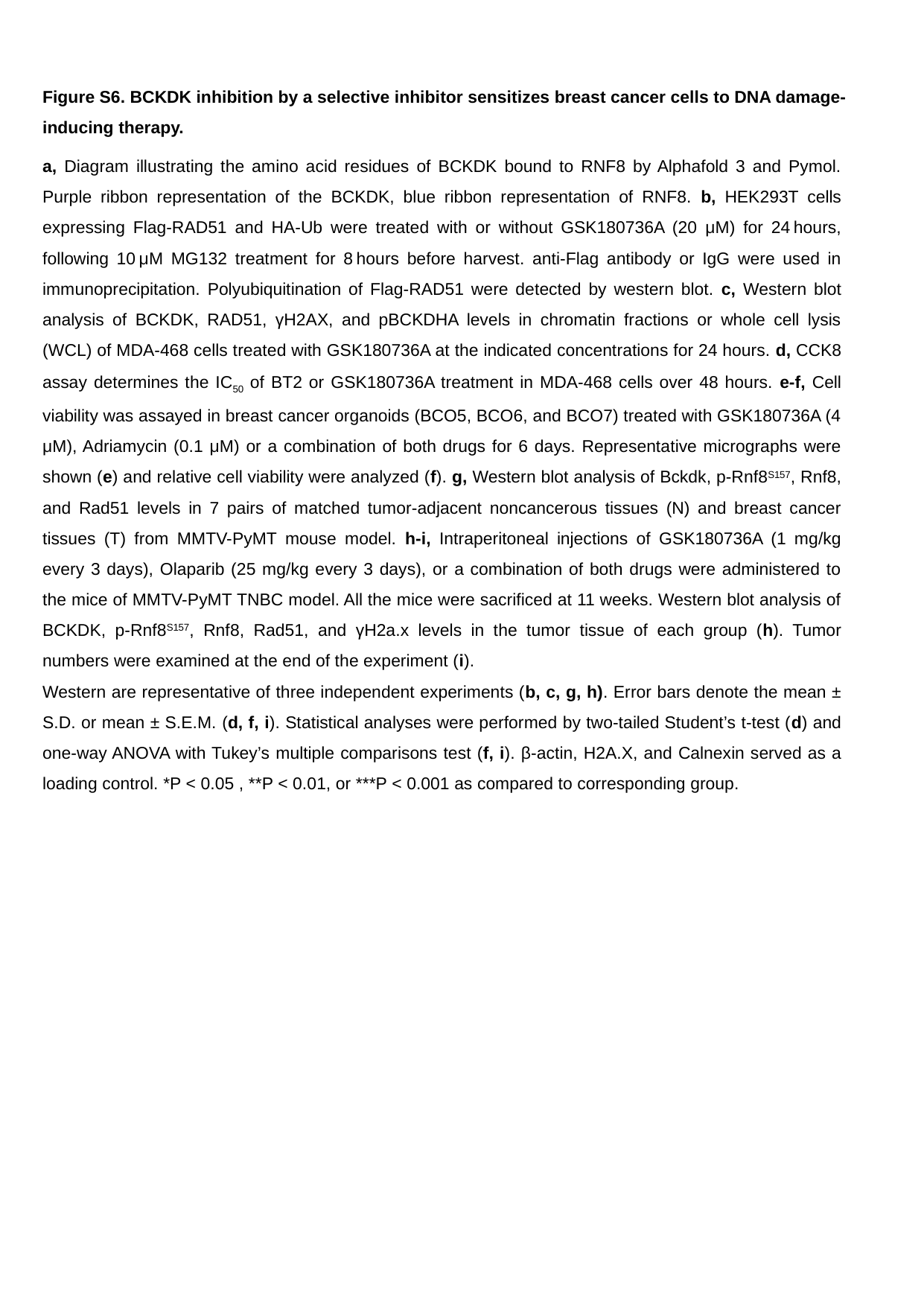

Figure S6. BCKDK inhibition by a selective inhibitor sensitizes breast cancer cells to DNA damage-inducing therapy.
a, Diagram illustrating the amino acid residues of BCKDK bound to RNF8 by Alphafold 3 and Pymol. Purple ribbon representation of the BCKDK, blue ribbon representation of RNF8. b, HEK293T cells expressing Flag-RAD51 and HA-Ub were treated with or without GSK180736A (20 μM) for 24 hours, following 10 μM MG132 treatment for 8 hours before harvest. anti-Flag antibody or IgG were used in immunoprecipitation. Polyubiquitination of Flag-RAD51 were detected by western blot. c, Western blot analysis of BCKDK, RAD51, γH2AX, and pBCKDHA levels in chromatin fractions or whole cell lysis (WCL) of MDA-468 cells treated with GSK180736A at the indicated concentrations for 24 hours. d, CCK8 assay determines the IC50 of BT2 or GSK180736A treatment in MDA-468 cells over 48 hours. e-f, Cell viability was assayed in breast cancer organoids (BCO5, BCO6, and BCO7) treated with GSK180736A (4 μM), Adriamycin (0.1 μM) or a combination of both drugs for 6 days. Representative micrographs were shown (e) and relative cell viability were analyzed (f). g, Western blot analysis of Bckdk, p-Rnf8S157, Rnf8, and Rad51 levels in 7 pairs of matched tumor-adjacent noncancerous tissues (N) and breast cancer tissues (T) from MMTV-PyMT mouse model. h-i, Intraperitoneal injections of GSK180736A (1 mg/kg every 3 days), Olaparib (25 mg/kg every 3 days), or a combination of both drugs were administered to the mice of MMTV-PyMT TNBC model. All the mice were sacrificed at 11 weeks. Western blot analysis of BCKDK, p-Rnf8S157, Rnf8, Rad51, and γH2a.x levels in the tumor tissue of each group (h). Tumor numbers were examined at the end of the experiment (i).
Western are representative of three independent experiments (b, c, g, h). Error bars denote the mean ± S.D. or mean ± S.E.M. (d, f, i). Statistical analyses were performed by two-tailed Student’s t-test (d) and one-way ANOVA with Tukey’s multiple comparisons test (f, i). β-actin, H2A.X, and Calnexin served as a loading control. *P < 0.05 , **P < 0.01, or ***P < 0.001 as compared to corresponding group.

## Slide 12
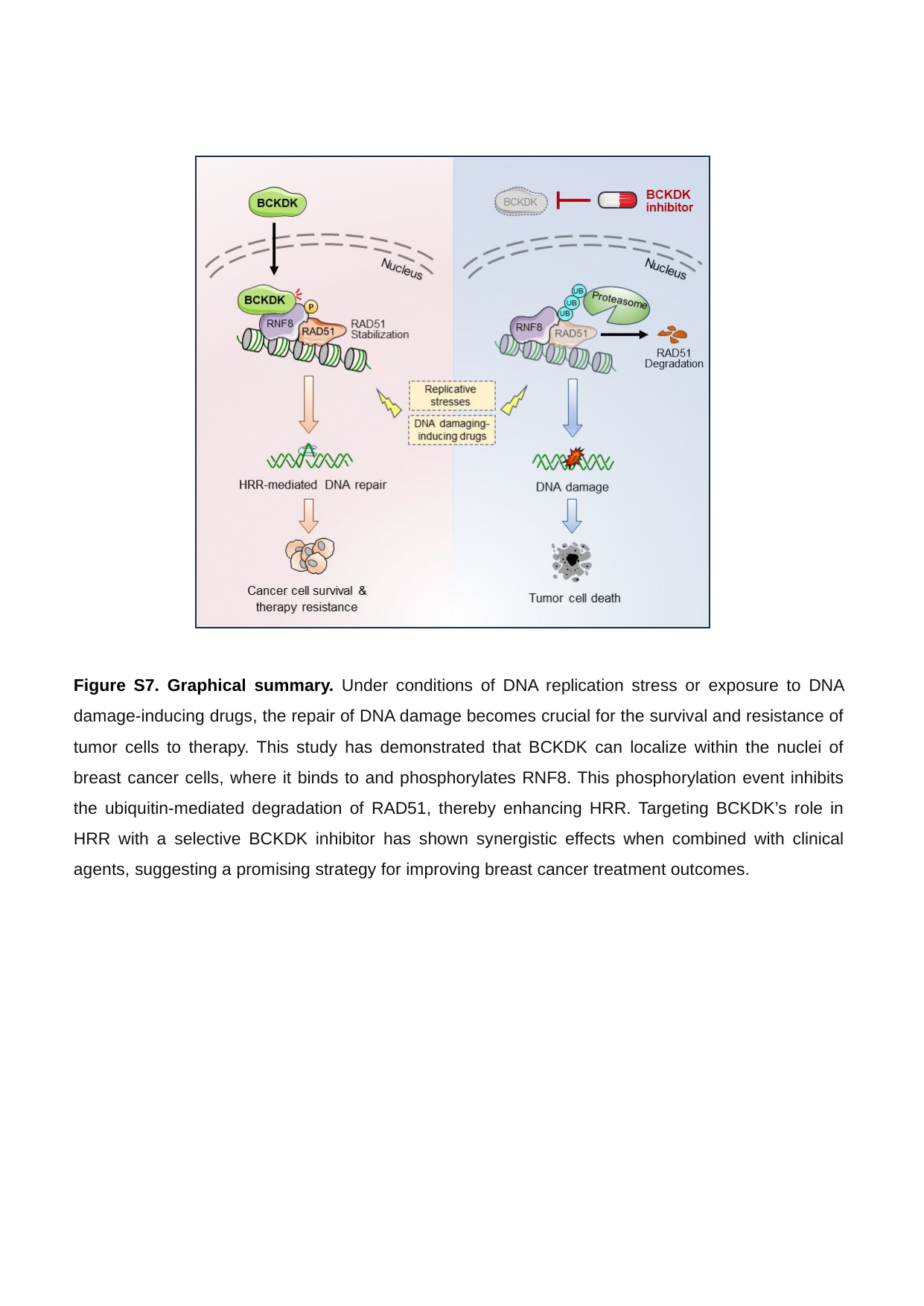

Figure S7. Graphical summary. Under conditions of DNA replication stress or exposure to DNA damage-inducing drugs, the repair of DNA damage becomes crucial for the survival and resistance of tumor cells to therapy. This study has demonstrated that BCKDK can localize within the nuclei of breast cancer cells, where it binds to and phosphorylates RNF8. This phosphorylation event inhibits the ubiquitin-mediated degradation of RAD51, thereby enhancing HRR. Targeting BCKDK’s role in HRR with a selective BCKDK inhibitor has shown synergistic effects when combined with clinical agents, suggesting a promising strategy for improving breast cancer treatment outcomes.
